# Alterations of the Gut Microbiome Associated to Methane Metabolism in Mexican Children with Obesity

**DOI:** 10.3390/children9020148

**Published:** 2022-01-24

**Authors:** Sofía Magdalena Murga-Garrido, Yaneth Citlalli Orbe-Orihuela, Cinthya Estefhany Díaz-Benítez, Ana Cristina Castañeda-Márquez, Fernanda Cornejo-Granados, Adrian Ochoa-Leyva, Alejandro Sanchez-Flores, Miguel Cruz, Ana Isabel Burguete-García, Alfredo Lagunas-Martínez

**Affiliations:** 1 Centro de Investigación sobre Enfermedades Infecciosas, Instituto Nacional de Salud Pública, Cuernavaca 62100, Mexico; sofiamurgaga@gmail.com (S.M.M.-G.); jcitla_oro@hotmail.com (Y.C.O.-O.); cediaz@insp.mx (C.E.D.-B.); cristy_acm@hotmail.com (A.C.C.-M.); aburguete@insp.mx (A.I.B.-G.); 2 PECEM, Facultad de Medicina, Universidad Nacional Autónoma de México, Ciudad de Mexico 04510, Mexico; 3 Departamento de Microbiología Molecular, Instituto de Biotecnología, Universidad Nacional Autónoma de México, Cuernavaca 62210, Mexico; fer.cornejog@gmail.com (F.C.-G.); adrian.ochoa@ibt.unam.mx (A.O.-L.); 4 Unidad Universitaria de Secuenciación Masiva y Bioinformática, Instituto de Biotecnología, Universidad Nacional Autónoma de México, Cuernavaca 62210, Mexico; alejandro.sanchez@ibt.unam.mx; 5 Unidad de Investigación Médica en Bioquímica, Centro Médico Nacional Siglo XXI, Instituto Mexicano del Seguro Social, Ciudad de Mexico 06720, Mexico; mcruzl@yahoo.com

**Keywords:** gut microbiome, childhood, obesity, methane, energy, dietary pattern

## Abstract

Gut microbiota is associated with the development of metabolic disorders. To study its association with childhood obesity, we performed a cross-sectional study with 46 children (6–12 years old). We collected fecal samples, food-frequency questionnaires (FFQs), and anthropometric measurements. Shotgun metagenomics were used to obtain the microbial taxonomic diversity and metabolic potential. We identified two dietary profiles characterized by complex carbohydrates and proteins (pattern 1) and saturated fat and simple carbohydrates (pattern 2). We classified each participant into normal weight (NW) or overweight and obese (OWOB) using their body mass index (BMI) z-score. The ratio of Firmicutes/Bacteroidetes and alpha diversity were not different between the BMI groups. Genera contributing to beta diversity between NW and OWOB groups included *Bacteroides rodentium*, *B. intestinalis*, *B. eggerthii*, *Methanobrevibacter smithii*, *Eubacterium* sp., and *Roseburia* sp. *B. rodentium* was associated with lower BMI and dietary pattern 1 intake. *Eubacterium* sp. and *Roseburia* sp. were associated with BMI increments and high consumption of dietary pattern 2. Methane and energy metabolism were found enriched in under-represented KEGG pathways of NW group compared to OWOB. Complex dietary and microbiome interaction leads to metabolic differences during childhood, which should be elucidated to prevent metabolic diseases in adolescence and adulthood.

## 1. Introduction

Obesity is a multifactorial disease in which increased energy intake is stored as fat [1,2]. It has become a global public health issue, particularly in countries like Mexico, where its prevalence has increased [3].

Disequilibrium of microbiota composition, known as dysbiosis, is common in many inflammatory non-communicable chronic diseases (NCCD) where obesity is included [4,5,6]. Remarkably, the Firmicutes-to-Bacteroidetes (F:B) ratio has been associated with disease occurrence [7,8,9]. Children with obesity compared to normal-weighted children have a higher F:B ratio [5,10,11]. In addition, the Firmicutes phylum has been associated with alterations in energy metabolism and positively correlated with energy harvest and fat mass accumulation, whereas the Bacteroidetes proportion has been correlated with loss of fat [8,12].

Bacterial genera, such as *Bacteroides*, *Roseburia*, *Bifidobacterium*, *Faecalibacterium*, and *Enterobacteria*, typically ferment carbohydrates into short-chain fatty acids (SCFA), which in turn bind themselves to G protein-coupled receptors expressed in the intestinal epithelium and adipose tissue [13,14]. Particularly, acetate and propionate can stimulate adipogenesis through GPCR43, whereas butyrate has primarily been associated with health benefits, such as the maintenance of colonic health by moderating cell growth and differentiation of colonic epithelial cells and by restoring intestinal permeability and repressing inflammatory responses [15]. Variations in carbohydrate, protein, and fat intake impact the structure of the microbial community, allowing specific bacterial types to be more prevalent [16,17,18]. Microbiota components and products interact with the host and affect many physiological processes, thus contributing to inter-individual variation [18]. Microbial metabolism can play important roles in energy extraction from the diet, which can lead to fat deposition and inflammatory processes [19].

Obesity has not only been linked to microbiota’s compositional shifts but also to functional alterations [20]. Comparing microbiomes of lean and obese individuals, Thingholm et al. found differences in functional capacity, including decreased superoxide reductase capacity, associated with obesity [20]. Phenotypic differences can be achieved with few gene differences between bacteria, heightening the importance of microbial metabolic potential parallel to taxonomic composition [21,22]. An altered proportion of genes encoding membrane transport functions; butyrate production; cofactor, vitamins, and nucleotide metabolism; and transcription process has been described in populations with obesity and diabetes [20,23,24]. Moreover, bacterial metabolites, such as methane, have been reported as modulators of host energy balance affecting caloric harvest [25]. Methanogens increase the capacity of polysaccharide-eating bacteria to digest polyfructose-containing glycans, leading to increased weight in mice models [26].

The gut microbiome has high inter-individual variability, and it is sensitive to environmental influences; therefore, it is essential to characterize it population-wise to study dysbiosis-associated diseases [27]. In the Mexican infant population, gut microbiome taxonomy, functional composition, and their dynamics are largely unknown; hence, we aim to investigate their characteristics in a group of children between the ages of 6 and 12 years old according to their dietary profile and body mass index. We found microbiome compositional differences involving essential taxa, methane, and energy metabolism between lean and overweight/obese urban children associated to dietary patterns. Since microbiota develops structure, establishes function, and matures throughout infancy and childhood, this is a promising time lap to promote health and prevent later-onset disease. 

## 2. Results

### 2.1. Overview of the Urban Children Population and Adiposity Classification

This study included a subsample of 46 individuals between 6 and 12 years old from a cross-sectional study previously reported [17]. Recorded individual information includes family history of obesity and overweight, biochemical blood parameters, dietary frequency questionnaire, and current infectious diseases along with phenotypic characterization, including values of age, sex, and body mass index (BMI).

All individuals were grouped into normal weight (NW) or overweight and obese (OWOB) using the World Health Organization (WHO) z-scores for childhood body mass index (BMI) adjusted by gender and age [28]. There were 21 female participants, of which 12 (57%) were classified as normal weight and 9 (43%) as overweight and obese. Meanwhile, from the 25 male participants, 15 (60%) were classified with normal weight and 10 (40%) with obesity (Table 1).

### 2.2. Dietary Profiles in the Urban Children Population

A food-frequency questionnaire (FFQ) was performed individually to calculate the daily intake of each food item (Supplemental Tables S1 and S2) [29]. From this information, we obtained two dietary patterns that were identified and interpreted as “pattern 1,” the healthier pattern characterized by the consumption of proteins and complex carbohydrates, and “pattern 2,” characterized by the consumption of saturated fat and simple carbohydrates (Supplemental Table S3). 

A score was obtained for each child that participated based on their reported diet in order to make an approximation on which pattern their diet is more likely to be characterized (Supplemental Table S4). After correcting the *p*-values for multiple comparisons, we did not find associations between the dietary patterns and the BMI of our population.

### 2.3. Microbiome Profile in Urban Children Population

**Figure 1:**
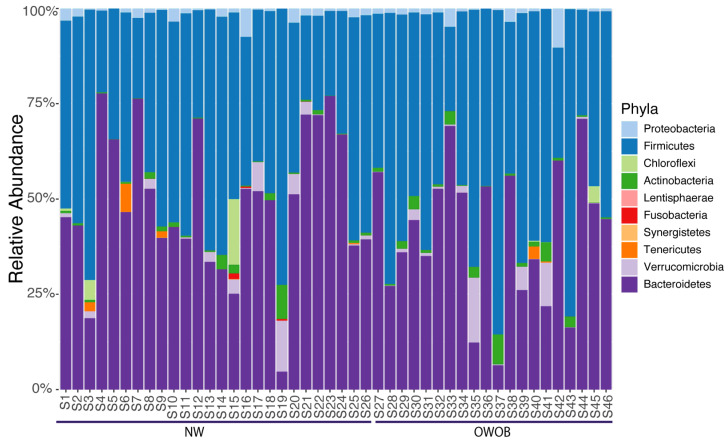
Phylum relative abundance of the 46 samples included in this study ordered by BMI.

Our high-quality microbiota reads were annotated using single copy phylogenetic marker gene (MG)-based operational taxonomic units (mOTUs) [30]. We found 917 taxa annotations across 46 samples further filtered by low abundance, leaving 875 taxa classified into ten phyla (Figure 1; Supplemental Table S5).

**Figure 2:**
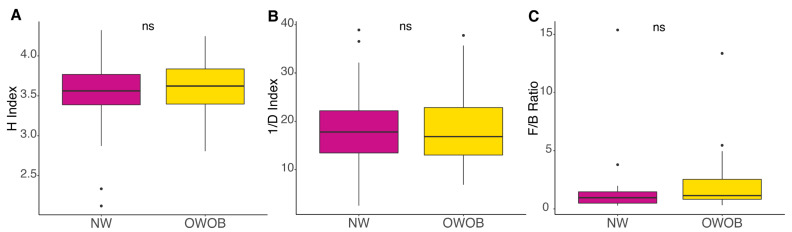
Alpha diversity, (**A**) Shannon Index (H), (**B**) Inversed Simpson diversity Index (1/D), and (**C**) Firmicutes-to-Bacteroidetes ratio (F/B) of normal-weight and obese-overweight groups. NW group is shown in magenta color and OWO in yellow. ns: *p* > 0.05. Pairwise comparisons were obtained using WRST and *t*-test.

The most abundant genera included *Bacteroides*, *Prevotella*, *Alistipes*, *Eubacterium*, *Ruminococcus*, *Akkermansia*, *Dialister*, *Clostridium*, *Faecalibacterium*, and *Barnesiella*. Given that microbiota dysbiosis favors an inflammatory status and impairs energy metabolism, we analyzed NW and OWOB group’s diversity [31,32]. Alpha diversity assessed by Shannon and inverse Simpson diversity index grouped by BMI status showed slightly higher values on the obese and overweight group although differences were not statistically significant (*p*-value > 0.05) (Figure 2A,B). The ratio between Firmicutes and Bacteroidetes was also higher in OWOB group although no statistical difference was found (*p*-value > 0.05) (Figure 2C).

We identified taxa that differ by BMI status performing a similarity of percentages analysis (SIMPER). The taxa that most contribute to beta diversity (Bray–Curtis) by abundance, which cumulatively explain 70%+ of the variation between NW and OWOB groups, include genera and species previously reported as physiologically important (Supplemental Table S6).

**Figure 3:**
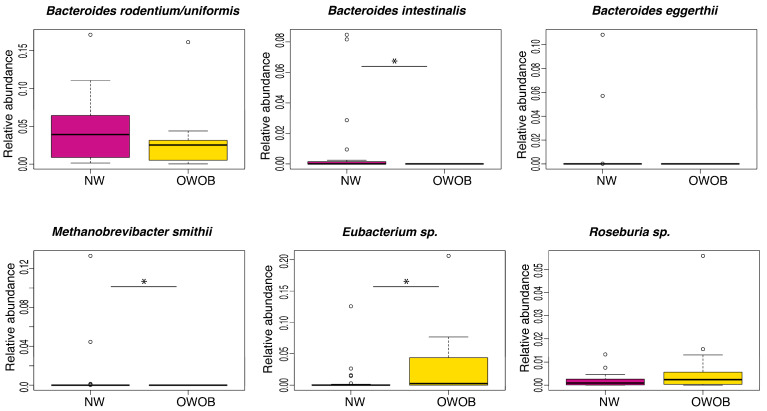
Relative abundance of taxa that showed difference in normal-weight (NW, magenta) group compared to overweight-obese (OWOB, yellow). Pairwise comparisons using Wilcoxon Rank-Sum Test adjusted by FDR: *Bacteroides rodentium/uniformis p*-value = 0.055, *Bacteroides intestinalis p*-value = 0.0012, *Bacteroides eggerthii p*-value = 0.073, *Methanobrevibacter smithii p*-value = 0.042, *Eubacterium* sp. *CAG:180 p*-value = 0.044, *Roseburia* species *p*-value = 0.098. *: *p* <= 0.05.

We found through the result of this analysis marginal statistical difference between the BMI groups in *Roseburia* sp. (*p*-value = 0.098), *Bacteroides rodentium* (*p*-value = 0.055), and *Bacteroides eggerthii* (*p*-value = 0.073), while *Bacteroides intestinalis* (*p*-value = 0.0012), *Methanobrevibacter smithii* (*p*-value = 0.042), and *Eubacterium* sp. (*p*-value = 0.044) abundance difference was statistically significant (Figure 3). Our results showed that normal-weighted children had increased relative abundance of *B. rodentium*, *B. intestinalis*, *B. eggerthii*, and *Methanobrevibacter smithii*, whereas overweight and obese children had incremented *Eubacterium* sp. and *Roseburia* sp.

We sought nutrimental vectors that fitted the beta diversity ordination to investigate linear relationships that contribute to the direction and strength of the bacterial diversity gradient. Remarkably, we found monounsaturated fatty acids (MUFAs) (r^2^ = 0.1513, *p*-value = 0.025), lipids (r^2^ = 0.1185, *p*-value = 0.057), saturated fatty acids (r^2^ = 0.1004, *p*-value = 0.080), trans fatty acids (r^2^ = 0.1062, *p*-value = 0.069), and lipid energy (kcal) (r^2^ = 0.1185, *p*-value = 0.057) as vectors in Bray–Curtis beta diversity.

Once we identified the taxa that differ between both BMI groups by similarity of percentage analysis, we performed an association analysis with our population macronutrient intake. In normal-weight-related taxa, *Bacteroides* appeared to increase with the consumption of carbohydrates (*B. rodentium* coef = 0.002, *p*-value = 0.08; *B. intestinalis* coef = 0.001, *p*-value = 0.046; *B. eggerthii* coef = 0.0013, *p*-value = 0.04) and fiber (*B. intestinalis* coef = 0.0014, *p*-value = 0.085; *B. eggerthii* coef = 0.0017, *p*-value = 0.04) and decrease with consumption of lipids (*B. rodentium* coef = −0.003, *p*-value = 0.06; *B. intestinalis* coef = −0.0016, *p*-value = 0.02; *B. eggerthii* coef = −0.002, *p*-value = 0.008) (Table 2). *M. smithii* relative abundance decreased with the protein intake (coef = −0.0035, *p*-value = 0.046). In overweight- and obesity-related taxa, *Roseburia*, increased with consumption of proteins (coef = 0.0017, *p*-value = 0.02), fiber (coef = 0.00074, *p*-value = 0.06), and overall healthier dietary pattern 1 (coef = 0.0029, *p*-value = 0.02), whereas *Eubacterium* abundance increased with sugar (coef = 0.0016, *p*-value = 0.007) and saturated fats (coef = 0.002, *p*-value = 0.007) intake and decreased with fiber (coef = −0.004, *p*-value = 0.022), polyunsaturated fats (coef = −0.003, *p*-value= 0.024), and dietary pattern 1 (coef = −0.01, *p*-value = 0.07).

We further sought associations between taxa abundance and BMI using z-scores stratified by each, low or high, dietary pattern consumption (Table 3). Higher relative abundance of *Eubacterium* sp. is associated with increased children’s BMI while having a high consumption of dietary pattern 2 (coef = 1.89, *p*-value = 0.019). A higher abundance of *Roseburia* sp. in addition to low dietary pattern 1 intake is associated with increased children’s BMI (coef = 15.93, *p*-value = 0.059). Furthermore, the effect of *B. rodentium* abundance on children’s BMI is based on the dietary pattern in a mirror-wise fashion, where a high consumption of proteins and complex carbohydrates (pattern 1) or low consumption of saturated fat and simple carbohydrates (pattern 2) results in the same outcome. *B. rodentium* abundance is associated with lower BMI in children with high dietary pattern 1 consumption (coef = −2.42, *p*-value = 0.013) and a low dietary pattern 2 intake (coef = −1.61, *p*-value = 0.053). 

**Figure 4:**
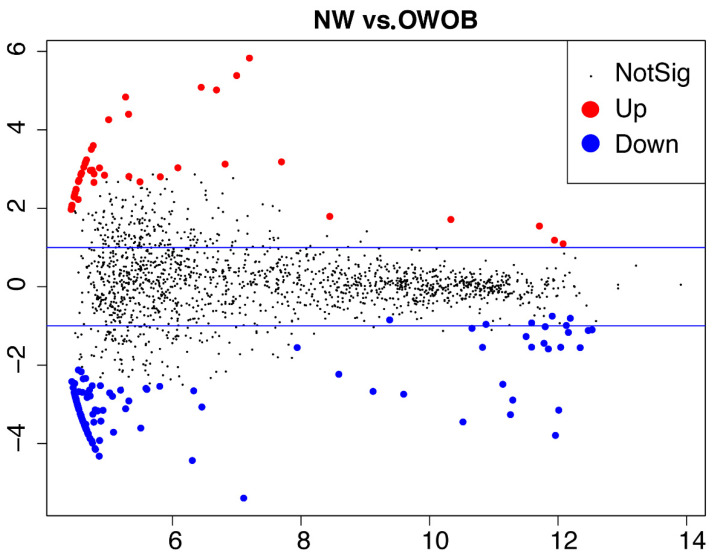
MA-plot showing differential microbiota genes representation (KOs) between normal-weighted vs. overweight and obese children. Differentially expressed genes (DEGs) *p* < 0.05; FDR adjusted *p* < 0.05. MA stands for the relationship between values of intensity (i.e., counts) and difference between the data (y-axis M = log ratio (log fold change) and x-axis A = mean, average of normalized counts).

Next, we carried out a gene-prediction analysis to investigate the microbiome functional potential. We assembled 86.06% of the high-quality reads, and 75% of the assemblies reported an N50 higher than 2000 bp. We found 1,582,297 nonredundant genes throughout all our samples. The average number of annotated genes per library was 123,326.67 ± 49,037.8 SD (Supplemental Figure S1), with a length average of 719.44 ± 60.04 pb. There was no gene richness difference between both BMI groups, but interestingly, we found 46 over-represented and 111 under-represented KOs (*p*-value abd FDR < 0.05) in the NW group compared to OWOB (Figure 4; Supplemental Table S7). 

**Figure 5:**
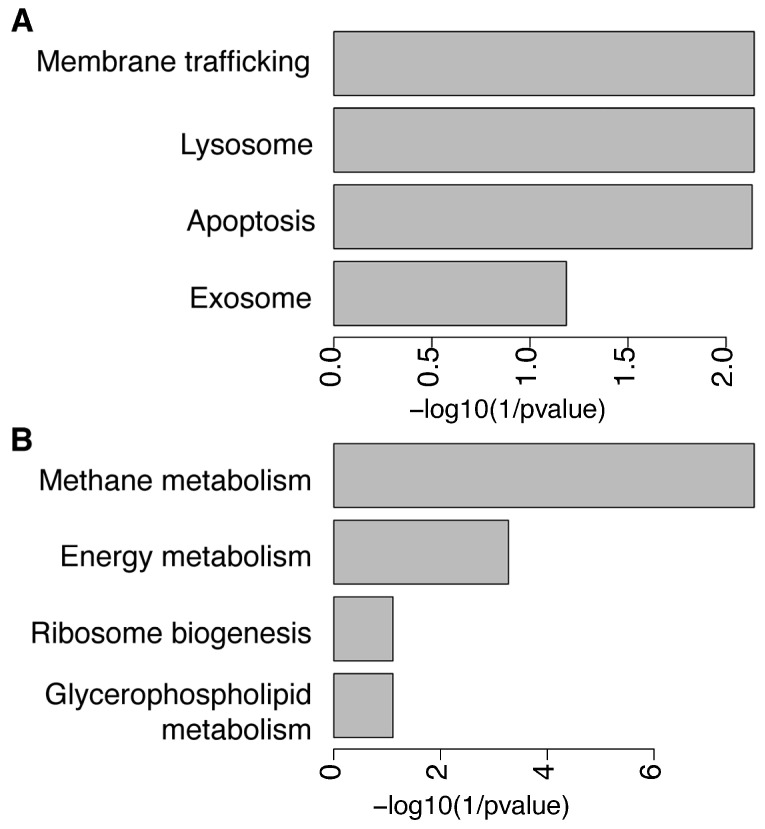
KEGG pathways over-represented (**A**) and under-represented (**B**) in NW group compared to OWOB.

We then investigated if each subset of differential KOs associated significantly with KEGG pathways to determine whether known biological functions were enriched in the microbiome of both BMI groups. We found 11 over-represented and 31 under-represented KEGG pathways further filtered by significant statistical difference (*p*-value) and false-discovery rate (Supplemental Table S8). The over-represented KEGG pathways in the NW group compared to the OWOB were membrane trafficking, lysosome, apoptosis (*p*-value and BH < 0.05), and exosome (*p*-value and BH < 0.1) (Figure 5A). Interestingly, methane metabolism, energy metabolism (*p*-value and BH < 0.05), ribosome biogenesis, and glycerophospholipid metabolism (*p*-value and BH < 0.1) appeared to be under-represented in NW gut microbiome group compared to OWOB (Figure 5B).

## 3. Discussion

Obesity and its complex etiology profoundly impact the quality of life [1]. Our data show that visceral abdominal fat accumulation in the OWOB group is higher than NW, which is the main predictor for the unhealthy obese phenotype [33]. In addition, most of the other criteria for metabolic syndrome, such as blood pressure, triglyceride, cholesterol, and glucose levels, were higher in the OWOB group. However, we did not find statistical differences, suggesting that this group might be at higher risk or at the beginning of metabolic impairment. 

Diet and microbiota play important roles in the development of obesity. Gut microbiota is a known biological factor with an effect on energy intake in the human body. Most abundant genera found in this study have also been reported in other studies [10,34,35]. Higher microbial diversity has been described in children with overweight [36,37]. Although not statistically significant in our study, probably due to our low sample size, alpha diversity also showed a higher trend in the OWOB group, suggesting that dietary and geographical effects could play an essential role, as previously reported [38,39]. Consistent with Bervoets et al., we also found an elevated although not statistically different F:B ratio in OWOB children compared to the NW group [10]. Taxa identified as more abundant in the OWOB group belong to the Firmicutes phylum, whereas taxa in NW group belong to the Bacteroidetes phylum, accordingly to the difference in Firmicutes: Bacteroidetes ratio between both groups. Previous studies have shown elevated counts of Firmicutes and fewer counts of Bacteroidetes in obese individuals when compared to those with normal weight, which might be associated with increased production of SCFAs and energy harvest [5]. Nevertheless, others have shown no difference in this phylum’s proportion between obese and non-obese groups, suggesting that F:B ratio is not a robust dysbiosis marker for obesity [40,41].

We used dietary patterns to analyze 46 urban children’s consumption aiming to capture the complexity of their eating habits. High-quality dietary pattern (defined by a balanced consumption of macronutrients in which energy is obtained by 20% proteins, up to 30% lipids, 50% carbohydrates) and high consumption of fruits, nuts, vegetables, whole grains, and yogurt has been inversely associated with weight gain and the risk of developing obesity [42]. In this study, pattern 1 was mainly characterized by high consumption of proteins and complex carbohydrates, which includes fiber. In general, carbohydrates serve as a major source of calories. Complex carbohydrates, derived from whole and unprocessed plant-based foods, are considered the healthier diet type and constitute a significant fraction of diet reaching the large intestine, where its effect is modulated by gut microbiome variations [43,44]. Fermentation of this complex fiber is performed by fibrolytic communities that usually belong to *Bacteroides*, *Roseburia*, *Ruminococcus*, and *Bifidobacterium* genus [43]. Our results showed increments of *Bacteroides* with the consumption of fiber and carbohydrates.

In this study, pattern 2 was characterized by the consumption of saturated fat and simple carbohydrates. Simple carbohydrates in diet usually provide calories without nutrients [45]. In addition, monosaccharides and disaccharides, both simple carbohydrates, can reach the large intestine when the host overfeeds with these sugars [46].

Similar to Murugesan et al. [47], in this study, we did not find dysbiosis but rather a different abundance of particular bacteria in gut microbiota between our groups. We identified three species of the *Bacteroides* genus as significantly more abundant in the NW group compared to OWOB, which is consistent with pattern 1 consumption. In this children population, the abundance of *Bacteroides rodentium*, along with high dietary pattern 1 and low dietary pattern 2 consumption, is associated with BMI decrement. Therefore, in the presence of *B. rodentium*, a leaner phenotype is produced when eating more proteins and complex carbohydrates or by consuming less saturated fat and simple carbohydrates.

Obesity has been associated with decreased abundance in *Methanobrevibacter smithii*, a gut microbiota methanogen archaea, which was also more abundant in the NW group [48]. In the gut, *M. smithii* plays a role in the production of ATP and in the remotion of fermentation end products, such as methanol and ethanol, produced by other bacteria [49,50]. *M. smithii* relative abundance decreased with protein intake, suggesting that complex carbohydrates are more likely to enhance its proliferation in the NW group.

On the other hand, consistent with a previous report, our OWOB group had a significantly higher abundance of *Roseburia* sp. [47]. We found an association between the abundance of *Roseburia*, low complex carbohydrates and proteins intake, and BMI increment. Moreover, carbohydrate intake, such as resistant starches, fructans, and fructose (a simple carbohydrate), has been associated with the *Roseburia-Eubacterium* abundance [51]. *Eubacterium* and *Roseburia*, among other genera, produce SCFAs from plant-vegetable products, which have been found increased in obese children, suggesting an elevated substrate utilization and increased energy harvesting by an obesogenic microbiome [5,37,52]. Increments of *Eubacterium rectale*, among other Firmicutes genera, has been related to obesity in humans and to higher adiposity levels, liver triglycerides, and glucose in mice fed with fructans and storage polysaccharides [34,44]. We found that the abundance of *Eubacterium* sp. and *Roseburia* sp. increases with sugar and saturated fats intake and proteins, respectively, suggesting that a Western-type diet might be driving their proliferation. Children’s dietary intake is complex and does not obey a single pattern [3]; a combination of macronutrients parallel to a more prevalent diet type might be potentiating substrate availability for some genus, thus explaining why we found that *Roseburia* sp. increases with the consumption of proteins, fiber, and overall healthier dietary pattern 1. Moreover, this is consistent with our finding associating *Eubacterium* sp. with BMI increment in children consuming the dietary pattern 2.

We found linear relationships between our population’s gut microbiota’s beta diversity and monounsaturated fatty acids (MUFAs), lipids, saturated fatty acids, trans-fatty acids, and lipid energy. These dietary lipids, long-chain saturated and unsaturated fatty acids, are absorbed in form of micelles by the intestinal enterocytes and further secreted systemically in chylomicrons [53]. Dietary saturated fatty acids are deleterious to metabolic health and have been correlated to obesity, hepatic steatosis, type 2 diabetes, and systemic inflammation mediated by IL-6 [54,55,56]. On the other hand, MUFAs have been mostly linked to anti-inflammatory states and constitute around 60% of fats present in Mediterranean diet and 36% of Western diet [53,57]. Higher MUFA consumption reduces saturated fatty acids and increases *Bacteroides*, *Prevotella*, and *Faecalibacterium* genera [58,59]. Our results showed decrements of *Bacteroides* associated with consumption of lipids probably belonging to saturated fatty acids. Lipid-fitted vectors on our samples’ diversity suggest a divergence prompted by diet.

Functional capacity differences parallel to compositional microbiome differences have been described between lean and obese individuals [20]. Accordingly, we found differences in KOs and functional pathway representation between lean (NW) and OWOB. Microbiome functional capacity of the OWOB group resulted in more than twice the number of represented KOs compared to NW. Housekeeping pathways related to membrane trafficking, lysosome, apoptosis, and exosome appeared enriched in the upregulated pathways in the NW group.

Methane and energy metabolism appeared significantly represented in the OWOB group. We also found a trending representation of ribosome biogenesis and glycerophospholipid metabolism in OWOB group. Energy metabolism has been predicted as a KEGG function in obese groups compared to lean ones [37]. Energy production can be increased by oxidative phosphorylation, glycolysis, and fatty acid synthesis from SCFA [60]. During fiber and sugar fermentation, H_2_ is produced and is dissipated by hydrogenotrophic microbes into other metabolites, such as methane (CH_4_), acetate, and H_2_S, some of the predominant intestinal gases [61]. Removal of H_2_ in the gut could allow more effective fermentation and upsurge SCFAs production, which increases energy absorption [50]. Even though *M. smithii* is the major methanogen responsible for the conversion of CO_2_ and H_2_ into CH_4_, methane production is also influenced by the amount and types of dietary substrates [61,62]. Methane excretion has been associated with the consumption of xylan and pectin, which are polysaccharides and a microbiota-accessible carbohydrate (MAC), respectively, in diet [63,64]. Furthermore, methane has been described as a gasotransmitter that slows gastrointestinal motility and as a biomarker in gut function alterations [61,62]. Lowering bowel transit promotes gut microbiome load and amplifies the time energy can be harvested, thus contributing to weight gain [65]. Furthermore, it has been shown that presence of methane and hydrogen on breath is associated with higher BMI and body fat percentage [25,65]. Our results support the hypothesis that methane metabolism is a potential modulator of host energy balance [66].

## 4. Conclusions

In the urban Mexican children population, proteins, simple and complex carbohydrates, and lipids seem to be dietary key factors driving major differences in BMI and microbiome effects on the host.

Taxa contributing to beta diversity difference between NW and OWOB groups appeared to be associated with dietary patterns. For example, *Eubacterium* and *Roseburia* genera were associated with increased BMI when the intake of saturated fats and simple carbohydrates was high and the intake of proteins and complex carbohydrates was low. On the other hand, *Bacteroides* genus was associated with decreased BMI during high consumption of complex carbohydrates and proteins.

The association of methane metabolism and obesity has been inconsistent across populations and studies [66]. In this study, we found the methane and energy metabolism of the gut microbiome in Mexican children with overweight or obesity enriched, which is most likely modulating energy balance harvested from the diet. This study contributes to the hypothesis that macronutrient intake modifies the intestinal gas profiles.

A major limitation of this study is that only two communities (NW vs. OWOB) were compared due to subsample size. Although these show organismal and functional differences, expanding this study to include an overweight group could give more information on the dynamics and transition of these differences. In addition, other limitations are related to food-consumption frequency. However, this nondifferential error is not likely to have an effect on the validity of our results. Further investigation is needed to stratify subjects using personalized predictions and to identify microbial biomarkers associated with the distinct dietary responses.

## 5. Materials and Methods

Sample and data collection: This cross-sectional study is part of a previously reported study [17] approved by the Research Committee of the National Institute of Public Health (INSP, No.1129). Ethical approval on the project was given by the INSP Commission of Ethics (CI:1129-No.1294) on 27 August 2012.

We randomly selected 48 samples from a biological bank of 1042 fecal samples. Two sample reads failed to annotate correctly against the taxonomy and functional reference databases, excluding them and leaving a sample size of n = 46. A food-frequency questionnaire and anthropometric measurements were performed after obtention of assent and informed consent from all participants and their parents or legal guardians. Children with a diagnosis of infectious or gastrointestinal diseases and those who had taken antibiotics for two months prior to the study were excluded.

### 5.1. Adiposity

The *who2007* function was used through the *RStudio* program to calculate z-scores of the body mass index (BMI) adjusted by age and sex in children between 5 and 19 years old [28]. We used the age-adjusted reference tables of weight, height, and BMI from the R package.

### 5.2. Dietary Patterns

Baseline dietary intake was measured using a validated food-frequency questionnaire (FFQ)—comprised of 11 food sections— that was filled in the presence of the parents or tutor of each participant [29]. The one hundred and seven food items contained in FFQ were consolidated into 27 food groups based on nutritional characteristics (Supplemental Table S1). According to the reported frequency of each item inside the groups (10 options ranging from never to => six times a day), the average of the daily consumption was calculated. The obtained factor was multiplied by the equivalent grams that conforms a portion based on the reports of the Mexican National Health and Nutrition Surveys (ENSANUT) [67] to obtain the quantity of grams that each individual consumes daily of each food. The number of grams or milliliters consumed of each of the 27 groups of foods was calculated to obtain the percentage of contribution of each group to the total daily consumption, which then was normalized using z-scores (Supplemental Table S2). After corroborating that the quantitative data were correlated using the corrplot R package (v 0.84), we performed a principal component analysis (PCA) that yielded two factors with an eigenvalue threshold of 2.67, which explain 23.57% of the total variance of the individual’s diet (Supplemental Figure S2). To perform a better interpretation of the components, an orthogonal rotation (varimax) was performed in order to redistribute the explained variance, obtain the most extreme weight factor, and to distinguish the components (Table 2). The patterns were defined based on loading factors > 0.35. The two dietary patterns were interpreted as pattern 1, the healthier pattern characterized by the consumption of proteins and complex carbohydrates, and pattern 2, characterized by the consumption of saturated fat and simple carbohydrates (Supplemental Table S3). Finally, a score of each factor per individual was calculated, giving each participant a value for each dietary pattern. The highest value was interpreted as the more likely type of diet consumed by a particular child (Supplemental Table S4).

### 5.3. Genomic DNA Extraction

DNA was extracted from 200 mg of each fecal sample using the QIAamp DNA Stool Mini Kit (Qiagen, Hilden, Germany). Bacterial lysis was performed using QIAamp kit buffers, proteinase K, and high-temperature incubation. Released genomic DNA was recovered through wash and purification columns. Isolated DNA was stored at −20 °C until further use.

### 5.4. Metagenomic Sample Processing

Pair-end libraries were elaborated from 1-ng DNA, and strands were enzymatically fragmented with ATM to 300–600bp and tagged with unique combinations of Nextera XT index (REF: 15032350, Illumina) in a barcode sample-specific fashion. PCR was carried out under the following conditions: initial denaturation for 3 min at 72 °C and 30 s at 95 °C, followed by 12 cycles of denaturation for 10 s at 95 °C, annealing for 30 s at 55 °C and elongation for 30 s at 72 °C, and a final elongation step for 5 min at 72 °C. The genomic amplification was recovered using magnetic beads washed with 80% ethanol. PCR products were resuspended in Nuclease-free water and quantified using Qubit dsDNA HS Assay kit (Thermo Fisher Scientific; Waltham, MA, USA). Furthermore, concentration and fragment length of libraries were assessed using a Bioanalyzer system (Agilent Technologies; Santa Clara, CA, USA). A final library for sequencing was created with equimolar ratios of libraries from each sample. The genomic pool for all 46 samples was sequenced throughout two independent runs by the USec from INMEGEN on Illumina NextSeq 500 platform to obtain 1,624,041,934,150-bases pair-end reads in total.

### 5.5. Metagenomic Analysis

The quality of the 1,624,041,934 raw reads was assessed using the program FastQC (version 0.11.8) [68] followed by preprocessing using Cutadapt (version 1.18) [69] with which barcodes, bad quality bases (<20 Phred score), and short-length fragments (<20 paired bases) were trimmed. The remaining 1,618,036,578 high-quality reads were subjected to host genome contaminating reads filtering by first mapping them independently to *homo sapiens* assembly (Ensembl release 95, GRCh38.dna.alt) using Bowtie2 (version 2.3.4.3) [70]. Following this, the 1,617,251,896 reads that did not map to the human genome were identified using Samtools view (version 1.9) [71], −f 4 was specified for unmapped, and −F 4 for mapped reads before regenerating *fastq* files containing either microbiome or human genome sequences (custom Perl script).

On the other hand, 86.06% of our previously preprocessed (high-quality bacteria) metagenomic reads were assembled using the metaspades.py script of the program SPAdes, for each library (version 3.13.0) [72]. Suggested k-mer lengths by the program developers were used to compute the assembly: −k 21,33,55,77. The quality of the assembly was assessed using the Quality Assessment Tool (Quast, version 5.0.2 Metaquast) [73,74]. Through all samples, between 12,419 and 148,377 contigs were assembled with lengths between 15,528,656 and 243,879,740 bases (132,282,896 average). The reported N50 values were between 848 and 7940 bases. Contigs with less than 500 bases were filtered out.

### 5.6. Taxonomic Annotation

The 1,617,251,896 high-quality microbiome reads were used for taxonomic annotation using the program mOTUs2 (version 2.5.1) [30]. A phylogenetic marker gene (MG)-based operational taxonomic units (mOTUs) strategy was perform to profile more than 7700 microbial species. We used the resulting profile, which reported relative abundance for each mOTU at phylum, genus, and species level for further analysis. Rare taxa, defined as taxa that do not have a count greater than 10 reads in at least one sample, were removed. A relative abundance proportion filter threshold was set to 0.01% (Supplemental Table S5).

### 5.7. Diversity Analysis

Alpha and beta diversity were assessed using the R packages Phyloseq (version 1.30.0) and Vegan (version 2.5–6) [75,76]. The data were assessed for normal distribution using the Shapiro–Wilk test to further compare normal weight against overweight and obese groups by either Wilcoxon Rank-Sum Test or *t*-test. Non-metric multidimensional scaling (nMDS) analyses were performed to evaluate distances between groups. Stress values obtained were <0.2 for Bray–Curtis and Jaccard beta diversities. To obtain a list of mOTUs which cumulatively explain more than 70% of the variation between groups, we used the Vegan R package function *simper* with 999 permutations (Supplemental Table S6). Wilcoxon Rank-Sum Test was used to evaluate differences between groups of each taxa obtained by *simper*. Continuous variables were fitted as vectors on the nMDS analysis using the *envfit* function of the Vegan package [76].

### 5.8. Functional Annotation

A protein-coding gene prediction was performed using the Prokaryotic Dynamic Programming Genefinding Algorithm (Prodigal, version 2.6.3), with the meta option and with closed ends, using the filtered contigs as input [77,78]. We followed the custom-made metagenomic assembly and annotation pipeline found in https://github.com/qijunz/DO_metagenomics/tree/master/pipeline (5 July 2021). The primary annotation of each file was concatenated into a single file of pooled predicted open reading frames (ORFs). Predicted genes were then compared in an all-against-all fashion in order to cluster nonredundant sequences and extract the representative sequences database using CD-HIT and -EST (version 4.8.1) [79]. The sequence identity threshold was set to 95% with a word length of 8, the alignment coverage for the shorter sequence was set to 90%, and the program was set to cluster into the most similar group that met the threshold. The collection of nonredundant predicted genes was used to build a Bowtie index, using Bowtie2 (version 2.3.4.3) [70] for the alignment of each sample in order to obtain the number of genes in each library (Supplemental Figure S1A,B). Predicted genes were annotated throughout the KEGG database in order to obtain a KEGG Orthology number for each ORF predicted. Annotation was performed using Kofam and HMMER/HMMSEARCH against a customize hidden Markov model database of procaryotes KOs [80]. Gene abundance was obtained using RSEM and normalized by transcripts per million (TMP) values, which were further analyzed using quasi-likelihoodF-test (Qlf) in edgeR (v. 3.28.1) in order to obtain differential presence of genes between BMI groups (logarithmic fold change over 1 and under −1, *p*-value and FDR < 0.05) [81,82]. We further ran the Fisher-enrichment test for each group of significantly differentiated genes obtained by Qlf and further filtered the results by *p*-value and FDR < 0.05 (Supplemental Tables S7 and S8).

### 5.9. Association Tests

We performed linear regressions of continuous variables running the general linear model (glm R function) adjusting by covariates or confounding variables, such as age, sex, family history of obesity, and physical activity. Association analysis between BMI z-scores and taxa stratified by low and high dietary profile was further adjusted by each dietary pattern, 1 or 2. Adjusting variables were identified when, individually, they modified the coefficient more than 10%. *p*-Values were adjusted using Bonferroni correction through multiple comparisons; statistical significance was considered when *p* < 0.01.

## Figures and Tables

**Table 1:** General characteristics by BMI status of children from Mexico City.

N = 46		BMI Status	
Characteristics		NW (*n* = 26)	OWOB (*n* = 20)
Gender	Female	12 (57%)	9 (43%)
	males	15 (60%)	10 (40%)
Age (years old)		8.03 ± 1.79	8.9 ± 2.1
Weight (kg) ****		25.46 ± 7.29	40.91 ± 12.71
Height (cm) *		127.77 ± 13	135.32 ± 12
Waist: Hip **		0.82 ± 0.037	0.86 ± 0.038
Glucose (mg/dL)		81.15 ± 12.2	81.85 ± 8.45
Triglycerides (mg/dL)		75.23 ± 26.2	103.75 ± 46.43
Cholesterol	HDL(mg/dL)	50.84 ± 10.04	50.85 ± 12.36
	LDL (mg/dL)	100.88 ± 28.68	110.55 ± 32.32
	Total (mg/dL)	152.96 ± 37.95	168.15 ± 42.16
TA	Systole (mean)	93.84 ± 10.27	98.85 ± 10.41
	Diastole (mean)	64.38 ± 9.15	65.12 ± 7.5
Physical activity (Mets)		342.01 ± 293.75	399.86 ± 441.33
Family history of overweight/obesity (%)		50%	70%

The data in this table are presented as mean ± standard deviation for continuous variables or percentage for categorical variables. Mean age was 8 years old for normal-weight group and almost 9 years for the group with overweight and obesity. Weight (*p*-value = 9.8 × 10^−6^), height (*p*-value = 0.02), and waist-to-hip ratio (*p*-value = 0.0022) resulted statistically different between both BMI classifications. Glucose, triglycerides, cholesterol, and blood pressure (BP) values were not statistically different although OWOB group showed increased mean values compared to NW. Pairwise comparisons using WRST and *t*-test were performed to compare data from both groups. ns: *p* > 0.05, *: *p* <= 0.05, **: *p* <= 0.01, ****: *p* <= 0.0001.

**Table 2:** Association analysis between taxa and macronutrient percentage intake.

N = 46	*Bacteroides rodentium*		*Bacteroides intestinalis*		*Bacteroides eggerthii*		*Methanobrevibacter smithii*		*Roseburia* sp.		*Eubacterium* spcag180	
	coef	*p*	coef	*p*	coef	*p*	coef	*p*	coef	*p*	coef	*p*
Carbohydrates (%)	0.002	0.08	0.001	0.046	0.0013	0.04	0.00009	0.9	−0.0004	0.14	−0.0003	0.8
Lipids (%)	−0.003	0.06	−0.0016	0.02	−0.002	0.008	0.0006	0.43	0.0002	0.54	0.001	0.33
Protein (%)	−0.0007	0.83	0.00005	0.97	0.0008	0.62	−0.0035	0.046	0.0017	0.02	−0.004	0.14
Sugars (%)	−0.001	0.1	−0.00009	0.74	−0.0003	0.19	0.00003	0.92	−0.00008	0.55	0.0016	0.007
Fibers (%)	0.001	0.42	0.0014	0.085	0.0017	0.04	−0.0012	0.19	0.00074	0.06	−0.004	0.022
Saturated fats (%)	−0.001	0.17	−0.0003	0.24	−0.0003	0.33	−0.0001	0.69	0.00002	0.86	0.002	0.007
monounsaturated fats (%)	−0.0001	0.92	0.0006	0.37	−0.0003	0.62	−0.0007	0.4	0.00013	0.73	−0.0018	0.25
polyunsaturated fats (%)	0.002	0.12	0.0006	0.35	0.0005	0.43	0.0005	0.49	0.000018	0.95	−0.003	0.024
Trans fat (%)	0.011	0.26	0.0021	0.63	0.0032	0.49	0.004	0.41	−0.0016	0.46	−0.003	0.7
Pattern 1	0.004	0.47	0.002	0.349	0.0036	0.18	−0.0036	0.22	0.0029	0.02	−0.01	0.07
Pattern 2	−0.008	0.14	0.00019	0.94	0.0002	0.92	0.0023	0.44	−0.0005	0.66	0.0009	0.87

Association analysis (linear regression) between taxa and macronutrient percentage intake adjusted by age, sex, and family history of obesity.

**Table 3:** Association analysis between taxa and BMI z-scores according to dietary pattern intake.

	*Bacteroides rodentium*		*Eubacterium* spcag180		*Bacteroides* *intestinalis*		*Methanobrevibacter smithii*		*Roseburia* sp.		*Bacteroides* *eggerthii*	
Dietary Pattern 1	coef	*p*	coef	*p*	coef	*p*	coef	*p*	coef	*p*	coef	*p*
low	−0.97	0.28	0.89	0.19	−1.93	0.29	−1.89	0.11	15.93	0.059	−108.5	0.91
high	−2.42	0.013	2.25	0.17	−2.65	0.16	−45.6	0.66	0.102	0.97	−1.72	0.16
Dietary Pattern 2	coef	*p*	coef	*p*	coef	*p*	coef	*p*	coef	*p*	coef	*p*
low	−1.61	0.053	−0.43	0.77	−1.12	0.57	−141.97	0.25	4.85	0.13	−2.02	0.45
high	−1.53	0.3	1.89	0.019	−3.1	0.12	−1.54	0.26	10.83	0.32	−2.77	0.11

Association analysis between taxa and BMI z-scores according to dietary pattern intake. The linear regression model was adjusted by age, sex, family history of obesity, and by each pattern.

## Data Availability

The data reported in this paper are accessible in the NCBI Short Read Archive (SRA) under accession ID PRJNA721692.

## Supplementary Materials

The following are available online at https://www.mdpi.com/article/10.3390/children9020148/s1, Figure S1: Number of genes predicted for each library; Figure S2: Correlation matrix between food groups; Table S1. Each of the 107 food items contained in the 27 defined groups surveyed in the FFQ; Table S2: Z-scores transformed daily intake percentage table for each food group; Table S3: Dietary patterns obtained through PCA factorial analysis and orthogonal rotation; Table S4: Dietary pattern score obtained for each child that participated based on their reported diet; Table S5: Relative abundance (>0.01%) of bacterial genera detected in fecal samples; Table S6: Taxa (at genus or specie level) that contribute the most to beta diversity variation between NW and OWOB groups (SIMPER); Table S7: KO representation analysis; Table S8: Enriched pathways as determined by Fisher’s test.

## Abbreviations

BH	Benjamini–Hochberg method
BMI	body mass index
BP	blood pressure
ENSANUT	Encuesta Nacional de Salud y Nutrición
F:B	Firmicutes-to-Bacteroidetes
FDR	false-discovery rate
FFQ	food-frequency questionnaires
KEGG	Kyoto Encyclopedia of Genes and Genomes
KO	KEGG Orthology
LPS	Lipopolysaccharides
mOTUS	(MG)-based operational taxonomic units
MAC	microbiota-accessible carbohydrates
MUFAs	monounsaturated fatty acids
NCCD	non-communicable chronic diseases
NW	normal weight
OWOB	overweight and obese
PCA	Principal Component Analysis
SCFA	short-chain fatty acids
SIMPER	similarity of percentages analysis
WHO	World Health Organization
