# Supplementary material for: Alterations of the Gut Microbiome Associated to Methane Metabolism in Mexican Children with Obesity
Source: Children (Basel). 2022 Jan 24;9(2):148. doi: 10.3390/children9020148 (PMC8870140; doi:10.3390/children9020148)
Supplement: Supplementary file 1 [file children-09-00148-s001.zip › SUPLEMENTAL FIGURE AND TABLES.pdf]

SUPLEMENTAL FIGURE AND TABLES

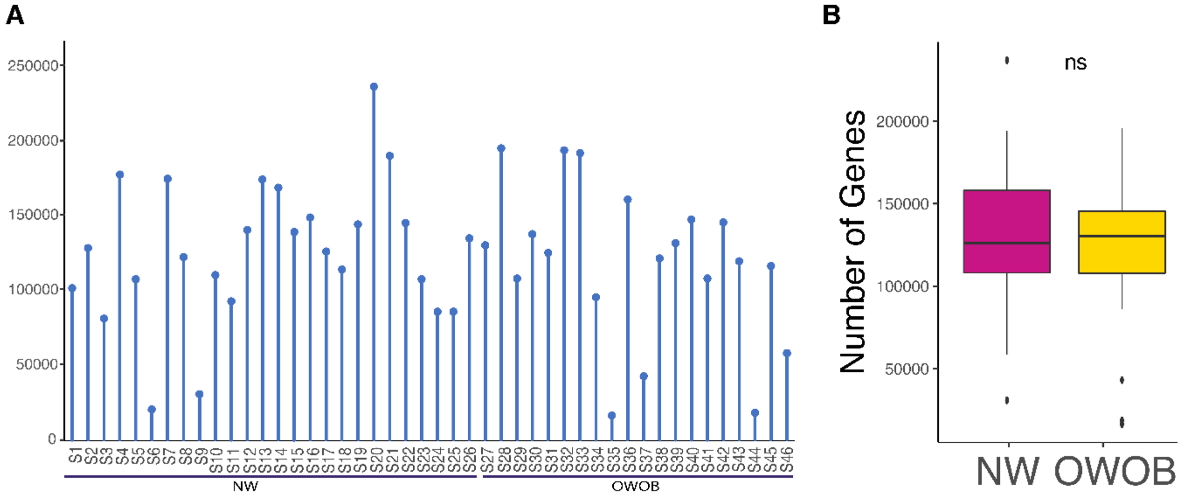

Figure S1: Number of genes predicted for each library (**A**) and pairwise comparison of number of genes predicted in NW vs OWOB (**B**).

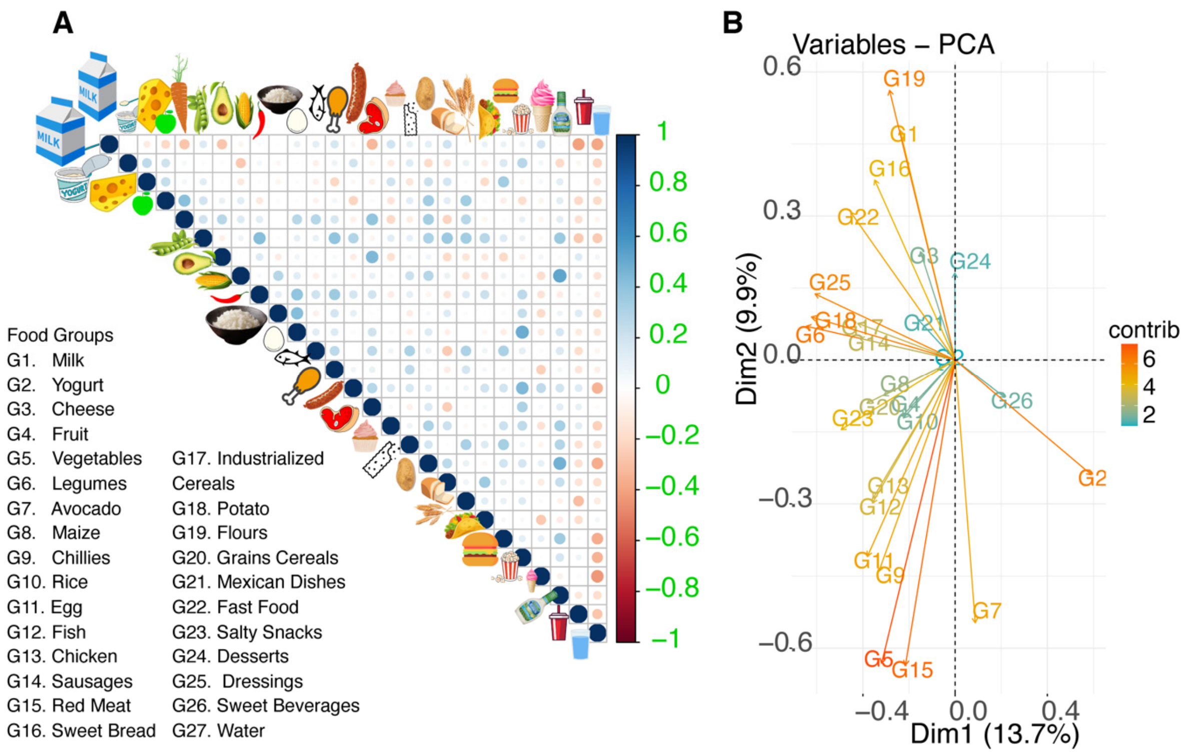

Figure S2: Correlation matrix between food groups that indicates relation between variables (**A**) and Principal Component Analysis (PCA) biplot of the 27 food group vectors were the principal component (PC) 1 explains 13.7% of total variance and PC2 explains 9.9% (**B**).

Table S1: Each of the 107 food items contained in the 11 sections surveyed in the FFQ.

	Original FFQ sections	Redefined food groups		Food items
**1**	Dairy	G1-Milk	1	whole milk (200 ml)
**1**	Dairy	G1-Milk	2	skim or light milk (200 ml)
**1**	Dairy	G24-Desserts	3	chocolate milk (200 ml)
**1**	Dairy	G3-Cheese	4	Oaxaca type cheese (slice)
**1**	Dairy	G3-Cheese	5	Manchego type cheese (slice)
**1**	Dairy	G3-Cheese	6	Cream cheese (15 ml)
**1**	Dairy	G3-Cheese	7	American cheese (slice)
**1**	Dairy	G2-Yogurt	8	Yogurth (250 ml)
**1**	Dairy	G24-Desserts	9	Petit Suisse (Danonino or Petizoo, 84 gr)
**1**	Dairy	G2-Yogurt	10	Yakult, Chamyto or Lalacult (fermented milk, 80 ml)
**1**	Dairy	G2-Yogurt	11	Activia Flora, Actimel or Svelty Gastro Protect (Probiotic yogurth, 255 ml)
**1**	Dairy	G24-Desserts	12	Ice cream or sorbet (100 gr)
**2**	Fruits	G4-Fruit	13	Banana
**2**	Fruits	G4-Fruit	14	Orange
**2**	Fruits	G4-Fruit	15	Orange juice (200 ml)
**2**	Fruits	G4-Fruit	16	Melon (slice)
**2**	Fruits	G4-Fruit	17	Watermelon (slice)
**2**	Fruits	G4-Fruit	18	Pineapple (slice)
**2**	Fruits	G4-Fruit	19	Papaya (slice)
**2**	Fruits	G4-Fruit	20	Peach
**2**	Fruits	G4-Fruit	21	Apple
**2**	Fruits	G4-Fruit	22	Pear
**2**	Fruits	G4-Fruit	23	Mango
**2**	Fruits	G4-Fruit	24	Tangerine
**2**	Fruits	G4-Fruit	25	Strawberries (1/2 cup)

**2**	Fruits	G4-Fruit	26	Grapes (1/2 cup)
**2**	Fruits	G4-Fruit	27	Plum (3)
**3**	Vegetables	G5-Vegetables	28	Tomato
**3**	Vegetables	G18-Potato	29	Potato
**3**	Vegetables	G5-Vegetables	30	Jicama
**3**	Vegetables	G5-Vegetables	31	Cucumber
**3**	Vegetables	G5-Vegetables	32	Carrot
**3**	Vegetables	G5-Vegetables	33	Lettuce (a leaf)
**3**	Vegetables	G5-Vegetables	34	zucchini or squash (1 cup)
**3**	Vegetables	G5-Vegetables	35	Nopal
**3**	Vegetables	G7-Avocado	36	Avocado (1/2)
**3**	Vegetables	G5-Vegetables	37	Broccoli (1 cup)
**3**	Vegetables	G9-Chillies	38	hot sauce (15 ml) or chlies
**3**	Vegetables	G9-Chillies	39	Canned chilies
**3**	Vegetables	G8-Maize	40	Corn
**4**	Legumes	G6-Legumes	41	Beans (200 gr)
**4**	Legumes	G6-Legumes	42	Peas (1/2 cup)
**4**	Legumes	G6-Legumes	43	lentils (200 gr)
**5**	Eggs, meats, and sausages	G11-Egg	44	Egg
**5**	Eggs, meats, and sausages	G14-Sausages	45	ham turkey or pork (slice)
**5**	Eggs, meats, and sausages	G13-Chicken	46	Chicken (1 piece)
**5**	Eggs, meats, and sausages	G15-RedMeat	47	Beef (125 gr)
**5**	Eggs, meats, and sausages	G15-RedMeat	48	Pork (125 gr)
**5**	Eggs, meats, and sausages	G12-Fish	49	Canned tuna (70 gr)
**5**	Eggs, meats, and sausages	G15-RedMeat	50	Pork rind
**5**	Eggs, meats, and sausages	G14-Sausages	51	Sausage (turkey or pork)
**5**	Eggs, meats, and sausages	G15-RedMeat	52	Bacon (slice)

**5**	Eggs, meats, and sausages	G13-Chicken	53	Liver (1 piece)
**5**	Eggs, meats, and sausages	G14-Sausages	54	Chorizo (1 piece)
**5**	Eggs, meats, and sausages	G12-Fish	55	Fish steak
**5**	Eggs, meats, and sausages	G12-Fish	56	Canned sardines
**6**	Mexican and typical dises	G21-MexicanDishes	57	Pork taco
**6**	Mexican and typical dises	G15-RedMeat	58	Barbacoa taco (sheep or lamb)
**6**	Mexican and typical dises	G21-MexicanDishes	59	Pastor taco (pork)
**6**	Mexican and typical dises	G21-MexicanDishes	60	Quesadilla, sope or gordita grilled
**6**	Mexican and typical dises	G21-MexicanDishes	61	Quesadilla, sope or gordita fried
**6**	Mexican and typical dises	G22-FastFood	62	French fries (1 portion)
**6**	Mexican and typical dises	G22-FastFood	63	Hamburger
**6**	Mexican and typical dises	G22-FastFood	64	Hot dog
**6**	Mexican and typical dises	G22-FastFood	65	Sandwich
**6**	Mexican and typical dises	G21-MexicanDishes	66	Torta
**6**	Mexican and typical dises	G22-FastFood	67	Pizza (slice)
**6**	Mexican and typical dises	G22-FastFood	68	Nuggets (3)
**6**	Mexican and typical dises	G21-MexicanDishes	69	Tamal
**6**	Mexican and typical dises	G21-MexicanDishes	70	Pozole (1 portion)
**7**	Cereals	G8-Maize	71	Maize tortilla
**7**	Cereals	G19-Flours	72	Flour tortilla (wheat)
**7**	Cereals	G19-Flours	73	White bread (slice)
**7**	Cereals	G20-GrainsCereals	74	Wholemeal bread (slice)
**7**	Cereals	G19-Flours	75	Bolillo or telera
**7**	Cereals	G16-SweetBread	76	Sweet bread
**7**	Cereals	G10-Rice	77	Rice (1 portion)
**7**	Cereals	G19-Flours	78	Soup (1 portion)
**7**	Cereals	G19-Flours	79	Pasta (1 portion)

**7**	Cereals	G20-GrainsCereals	80	Oatmeal (1 cup)
**7**	Cereals	G17-IndCereal	81	Cereal without sugar (1 portion)
**7**	Cereals	G17-IndCereal	82	Cereal with sugar (1 portion)
**7**	Cereals	G20-GrainsCereals	83	Cereal with fiber (1 portion)
**7**	Cereals	G16-SweetBread	84	hot cake (1 piece)
**7**	Cereals	G23-SaltySnacks	85	Saltines (1 personal package)
**8**	Beverages	G26-SweetBeverages	86	Cola soda (355 ml)
**8**	Beverages	G26-SweetBeverages	87	Soda (355 ml)
**8**	Beverages	G27-Water	88	Natural water (200 ml)
**8**	Beverages	G26-SweetBeverages	89	Water with natural fruit (200 ml)
**8**	Beverages	G26-SweetBeverages	90	Water with sweeteners (200 ml)
**8**	Beverages	G24-Desserts	91	Milkshake (200 ml)
**8**	Beverages	G26-SweetBeverages	92	Industrialized fruit juice (250 ml)
**8**	Beverages	G26-SweetBeverages	93	Sports drink (250 ml)
**9**	Sweets and desserts	G16-SweetBread	94	Cake (slice)
**9**	Sweets and desserts	G24-Desserts	95	Jam and condensed milk candy (15 ml)
**9**	Sweets and desserts	G24-Desserts	96	chocolate bar
**9**	Sweets and desserts	G23-SaltySnacks	97	Peanuts (1 portion)
**9**	Sweets and desserts	G23-SaltySnacks	98	Fried snacks (1 portion)
**9**	Sweets and desserts	G16-SweetBread	99	cupcake with creamy filling
**9**	Sweets and desserts	G16-SweetBread	100	Cookies
**9**	Sweets and desserts	G23-SaltySnacks	101	Popcorn
**9**	Sweets and desserts	G24-Desserts	102	Hot tamarind candy
**9**	Sweets and desserts	G17-IndCereal	103	Cereal bar
**10**	Fats	G25-Dressings	104	Butter (15 ml)
**10**	Fats	G25-Dressings	105	Dressing (15 ml)
**10**	Fats	G25-Dressings	106	Mayonnaise (15 ml)

**11**	Supplements	NA	107	Vitamins

Table S2: Z-scores transformed daily intake percentage table for each food group.

SampleID	G1-Milk	G2-Yogurt	G3-Cheese	G4-Fruit	G5-Vegetables	G6-Legumes	G7-Avocado	G8-Maize	G9-Chillies	G10-Rice	G11-Egg	G12-Fish	G13-Chicken	G14-Sausages	G15-RedMeat	G16-SweetBread	G17-IndCereal	G18-Potato	G19-Flours	G20-GrainsCereal	G21-MexicanDish	G22-FastFood	G23-SaltySnacks	G24-Desserts	G25-Dressings	G26-SweetBecera	G27-Water
S117	0.309	0.900	0.193	0.436	0.813	0.549	0.487	1.313	0.940	0.163	0.234	-0.401	-0.067	-0.342	-1.285	-0.175	-0.243	0.501	-0.756	0.134	-0.913	0.679	-0.013	-0.708	0.323	1.130	-1.133
S134	-1.630	-0.882	-0.668	0.444	0.899	-0.126	-0.238	1.151	1.260	0.069	0.137	-0.775	-0.182	-0.667	-0.335	-0.783	-1.037	-1.076	-1.117	-0.840	0.533	0.578	-0.498	-0.825	-0.030	0.867	0.725
S179	0.272	0.192	-0.244	-0.428	-1.517	-0.980	-0.712	-0.516	-0.699	-0.075	-0.010	-1.242	-0.356	-0.768	-0.965	2.335	0.312	0.234	-0.364	-0.147	-0.710	-1.242	-0.153	-0.332	-0.879	-0.666	1.199
S201	-1.387	-0.039	0.532	1.315	2.092	0.745	0.178	-0.084	-0.625	-1.308	-0.515	-0.441	0.910	1.057	-0.716	-0.163	0.540	1.401	0.776	2.039	-0.149	-0.575	0.327	0.109	0.589	-0.961	0.526
S329	-0.015	0.229	0.055	-0.539	-0.979	-0.163	2.723	0.495	0.931	-0.043	-0.853	-0.296	-0.317	-0.425	0.700	0.244	-0.388	0.269	-0.031	-0.634	-0.815	-0.952	-0.338	-0.354	-0.099	-0.720	1.274
S339	0.341	0.474	0.199	-0.501	-1.098	-1.112	3.326	0.647	-0.651	-1.067	-0.782	1.199	-0.060	-0.400	-0.775	-0.753	1.617	-0.609	0.236	-0.731	-0.453	-0.091	-0.362	0.544	0.180	-1.166	0.903
S402	0.480	-0.041	1.408	1.790	1.345	1.239	-0.876	0.398	-0.593	2.366	0.346	-0.686	0.065	-0.529	-1.474	-0.929	-1.277	0.622	2.014	1.473	0.337	-1.212	-0.633	-0.538	0.095	-1.360	-0.158
S573	-0.260	0.561	-0.774	-0.955	-0.301	-0.485	-0.876	-1.037	-0.066	1.464	-0.812	0.831	-0.758	1.516	-0.277	-0.214	1.254	-0.642	1.245	1.096	2.155	2.735	-0.109	-0.538	-0.879	0.802	-0.621
S578	0.342	-0.503	-0.761	-0.193	1.241	3.539	0.852	0.264	4.358	-0.701	-0.029	1.735	2.677	1.416	1.491	-0.271	1.447	-0.467	-0.843	2.050	2.425	-0.583	-0.354	-0.446	-0.130	-0.365	-0.553
S847	0.241	0.818	-0.632	0.224	-0.631	1.424	0.769	0.596	0.547	1.954	0.546	0.253	3.162	-0.484	-1.471	0.203	1.423	0.842	0.367	0.230	0.248	0.146	-0.196	-0.506	0.190	0.661	-1.013
S865	0.954	-0.523	-0.664	1.251	-0.777	1.008	0.867	-0.173	-0.699	0.574	0.655	0.165	0.432	-0.817	-0.480	-0.503	-0.846	-0.461	2.331	-0.879	2.402	-0.921	-0.450	-0.789	1.252	-1.329	0.139
S884	0.912	-0.741	-0.875	1.339	0.643	1.151	-0.017	-0.353	-0.487	-0.704	-0.965	0.145	-1.120	0.386	-0.013	0.597	-0.378	2.669	1.717	0.396	1.923	-0.951	0.478	0.139	-0.719	-0.398	-1.135

S1531	0.636	-0.497	0.465	-1.318	-1.332	-0.625	-0.876	-1.048	-0.699	-0.761	-0.714	-0.282	-0.526	0.001	-0.684	-0.837	0.475	-0.841	0.058	1.526	-0.699	1.011	0.074	2.923	-0.879	0.585	-1.051
S1708	2.604	-0.689	-0.367	-0.437	-0.283	0.590	-0.876	-0.486	-0.029	-0.353	-0.296	-0.763	0.465	-0.575	-1.308	-0.705	0.145	-0.078	0.724	-0.879	-0.113	-0.477	-0.308	0.615	-0.795	-0.094	-1.216
S1914	-1.100	-0.149	-1.034	0.042	-0.689	0.007	-0.210	-0.635	-0.438	0.129	-0.795	-0.574	0.175	-0.636	0.725	-0.380	0.578	-0.623	1.237	-0.601	0.990	0.291	-0.183	-0.835	0.892	-0.834	1.679
S1922	-0.264	-0.501	3.361	-0.388	-0.408	2.757	-0.510	0.332	-0.556	-1.179	-0.351	-0.790	-0.760	0.783	-0.255	3.540	-0.870	1.951	2.507	-0.879	-1.026	2.440	0.109	-0.622	3.086	0.407	-0.624
S1969	-0.194	-1.129	-0.381	1.210	-0.608	-0.456	-0.876	3.713	-0.699	1.570	-0.314	0.241	-0.716	0.670	-0.584	0.480	3.429	2.076	-0.159	-0.879	-0.832	1.393	-0.065	0.382	3.329	1.041	-1.712
S1972	0.163	2.883	-0.211	-0.980	-0.254	0.082	-0.876	-0.553	1.784	2.138	2.260	-0.679	-0.491	3.261	1.597	0.326	-0.488	0.109	-0.112	-0.093	0.097	-0.370	5.667	0.235	-0.187	0.816	-1.712
S2146	-0.970	-1.191	-0.490	-0.722	-0.166	0.540	1.050	-0.048	1.617	-0.631	0.858	-0.798	-1.032	-0.850	-0.582	-0.873	0.185	-0.389	0.836	0.837	1.028	-0.737	0.375	-0.556	-0.402	-0.829	1.969
S2148	-0.887	0.337	2.760	2.651	0.079	0.305	-0.054	-0.058	0.546	0.467	0.546	0.174	-1.152	-0.517	-0.045	-0.726	0.343	0.841	0.501	1.889	-0.727	1.147	-0.014	1.298	-0.294	-0.473	-1.014
S2213	1.331	-0.379	-0.712	-0.379	1.966	0.079	0.107	-0.025	-0.630	-0.962	0.901	4.291	0.723	-0.627	1.649	1.809	1.533	1.229	-1.288	2.160	-0.497	2.144	2.001	-0.343	2.223	-1.114	-0.877
S2216	-0.570	-0.810	-0.454	-0.796	1.775	0.526	1.235	1.852	1.053	2.879	4.162	0.863	0.915	0.129	1.637	-0.248	-0.582	-0.316	-0.516	-0.098	-0.674	-0.619	1.438	-0.618	1.441	-0.580	0.530
S2221	2.247	-1.090	-0.997	-0.815	-0.470	-0.430	1.538	-0.565	1.129	-0.459	1.398	0.379	1.311	-0.606	-1.201	0.110	0.555	-0.197	-0.223	-0.472	-0.729	-0.834	-0.483	-0.787	-0.879	-1.485	0.851
S2269	0.396	0.531	0.241	0.119	1.457	-0.557	0.538	-0.622	-0.649	0.219	0.291	0.772	-1.250	-0.479	0.414	0.215	-1.011	0.562	0.005	-0.261	-0.747	-0.312	-0.657	2.467	-0.638	-0.341	-1.111
S2270	-0.266	-0.670	1.494	-0.409	-0.893	-0.410	0.148	-0.929	1.104	0.722	1.361	0.827	0.612	-0.113	0.549	1.627	-0.500	1.127	0.162	-0.848	0.299	0.772	0.481	-0.342	-0.847	2.250	-1.277
S2289	-0.465	-0.885	-0.001	-0.495	1.786	-0.891	-0.552	1.857	-0.699	1.138	-0.458	-0.274	1.118	-0.679	2.737	-1.042	-1.131	1.592	-0.455	-0.741	-0.044	0.873	-0.378	-0.662	-0.633	-0.450	0.694
S2345	0.902	0.560	-1.021	-1.173	-0.675	1.687	-0.876	-0.500	-0.639	-1.007	-0.658	-0.510	0.392	2.279	0.386	-0.309	-1.217	-0.473	-0.264	-0.879	-0.356	1.302	-0.146	3.370	0.261	-0.435	-1.399

S2429	0.568	-1.078	-0.951	0.763	-0.008	0.064	-0.876	0.361	-0.699	-0.142	0.921	-0.905	-0.438	1.178	1.250	-1.004	0.628	1.888	0.038	0.393	0.102	-0.559	-0.381	0.288	1.510	-1.422	0.351
S2452	-1.192	-0.988	-0.836	-1.286	-0.906	-0.324	-0.279	-0.485	-0.632	-0.019	0.047	-0.643	-0.288	-0.550	-1.069	-0.487	-0.982	-0.912	-0.762	-0.754	-1.070	-1.062	-0.720	-0.722	-0.713	1.936	1.331
S2468	0.642	-0.598	-0.338	-0.406	0.123	-0.885	-0.653	-0.267	-0.699	0.375	-0.994	-0.280	0.190	-0.653	-0.647	1.150	-1.030	-0.840	-0.379	-0.710	-0.995	-0.985	-0.504	-0.649	0.207	0.988	-0.057
S2527	-0.263	1.326	-0.260	0.963	-0.401	0.692	-0.876	-0.487	-0.573	-0.201	-0.905	0.024	-0.509	-0.257	-0.364	0.592	1.312	-1.090	-0.345	2.349	0.135	-0.323	-0.286	-0.771	-0.879	-1.173	0.902
S2593	-0.327	-0.158	-0.183	0.275	-0.027	-0.423	2.158	1.611	-0.699	-0.242	-0.919	-0.632	-0.559	-0.625	-0.092	-0.488	-1.178	-0.758	-1.352	-0.673	-0.933	-0.593	-0.539	0.079	-0.008	0.637	0.176
S2600	-0.731	2.424	-0.802	1.639	1.107	0.130	-0.876	-0.390	-0.607	-0.730	-0.683	-0.139	2.103	-0.517	-0.689	-0.875	1.264	-0.501	-0.365	0.610	-1.032	0.608	-0.262	-0.523	-0.650	-0.389	0.032
S2601	-0.152	-1.072	-0.986	-1.283	-0.972	-1.092	-0.486	2.292	-0.306	-0.828	-0.784	-1.094	-1.272	-0.507	0.624	0.571	-0.042	-0.994	2.407	-0.704	0.385	-0.647	0.812	-0.432	-0.119	2.158	-0.554
S2608	0.845	1.852	1.715	-1.496	-1.251	-0.073	-0.036	-0.790	0.694	0.504	-0.973	-0.206	0.347	1.085	-0.671	-0.656	0.343	-1.053	0.119	-0.647	-0.676	0.711	-0.472	-0.106	0.290	0.023	0.070
S2627	-1.110	-1.050	-0.768	-0.849	-0.986	-0.932	-0.638	-0.204	-0.494	0.490	-0.675	0.102	0.330	3.106	1.212	1.309	0.398	-0.819	-0.836	-0.827	0.762	-0.668	-0.341	0.512	-0.286	1.338	0.056
S2632	-1.019	0.285	1.483	-1.180	0.017	-0.777	0.574	-1.143	-0.669	-0.798	-0.752	0.449	-1.235	-0.134	-0.679	-1.061	-0.126	-1.066	-1.079	-0.791	1.784	0.843	-0.521	-0.718	-0.722	-0.507	1.982
S2663	-1.980	0.844	0.673	1.799	0.772	-0.405	-0.044	-0.680	-0.699	-0.723	-0.975	-0.215	0.328	0.196	1.387	0.164	-0.406	-0.820	-0.412	0.833	-0.127	-0.731	-0.338	-0.756	-0.467	1.308	0.054
S2680	0.200	0.008	0.134	0.305	1.304	-0.614	0.423	-0.669	0.284	-0.851	0.163	-0.123	-1.299	-0.732	-0.686	-0.600	-0.777	0.423	-1.074	-0.588	-0.821	-0.742	-0.261	2.277	-0.457	-0.577	-0.333
S2726	-1.078	-1.202	-0.594	-0.651	0.084	-0.582	0.487	-1.067	0.530	-0.829	-0.784	2.852	-0.067	-0.495	1.085	-1.016	-0.941	-0.611	-1.142	-0.279	-0.921	-0.250	-0.540	-0.275	-0.584	0.116	1.760
S2817	-1.015	0.667	0.923	1.084	0.818	-0.137	-0.148	-0.744	0.434	-0.796	0.337	-0.343	0.055	-0.202	1.015	-0.269	-0.022	0.613	-0.509	-0.834	-0.651	0.137	0.043	0.430	0.405	0.261	-0.166
S2846	2.195	1.731	0.987	0.844	-0.817	-0.541	-0.624	-0.145	0.521	-0.688	0.677	-0.058	0.565	-0.857	-0.302	0.324	0.548	-0.800	-0.035	-0.300	0.799	1.283	-0.315	-0.457	-0.879	-1.041	-1.120

S2883	-0.828	1.303	0.782	0.714	0.765	-1.256	-0.876	-0.305	-0.699	-0.538	1.151	-0.142	-0.919	-0.592	0.263	-0.687	-0.942	-1.147	-1.112	-0.811	-0.741	-0.732	-0.457	-0.639	-0.811	0.424	0.614
S2927	-0.560	0.641	-0.856	-0.479	-0.789	-0.900	-0.876	-0.690	-0.699	-1.009	-0.333	-0.717	-1.491	-0.552	-1.236	-1.120	-0.421	-0.812	-0.976	-0.701	0.450	-1.019	-0.568	-0.048	-0.787	1.493	0.455
S2967	0.791	-0.722	0.085	-0.897	-0.287	-0.855	-0.876	-0.497	-0.699	-0.041	0.024	-0.876	-0.315	-0.240	0.253	1.741	-0.266	0.271	-0.172	-0.590	1.621	-0.150	0.240	-0.496	0.156	-0.698	0.530
S2971	0.890	-0.980	-0.632	-0.155	-1.559	-1.083	-0.876	-0.686	-0.699	0.466	-0.683	-0.417	0.301	-0.638	-0.090	-0.164	-1.229	-0.824	-0.600	1.056	-1.022	-0.756	-0.204	0.727	-0.777	0.166	0.033

Table S3: Dietary patterns obtained through PCA factorial analysis and orthogonal rotation.

Food Groups	Foods Included	Pattern 2: Saturated Fat and Simple Carbohydrates	Pattern 1: Proteins and Complex Carbohydrates
**1. Milk**	Skim and Whole Milk		
**2. Yogurt**	Yogurt Flavoured, Yakult, Yogurt Actiregularis®, Yogurt Natural		
**3. Cheese**	Oaxaca, Manchego, American, and Cream Cheese		
**4. Fruit**	Strawberry, Tangerine, Orange, Banana, Peach, Apple, Pear, Grapes, Mango, Plums, Pineapple, Papaya, Melon, Watermelon, Orange Juice		
**5. Vegetables**	Tomato, Carrot, Cucumber, Jicama, Lettuce, Zucchini, Nopal, Broccoli		0.5806
**6. Legumes**	Beans, Peas, Lentils		0.527
**7. Avocado**	Avocado	-0.41	
**8. Maize**	Corn Tortilla, Corn		
**9. Chillies**	Canned Chillies, Hot Sauce		0.5908
**10. Rice**	Rice		
**11. Egg**	Eggs		0.5042
**12. Fish**	Fish, Sardines, Tuna		0.5541
**13. Chicken**	Chicken and Liver Chicken		0.5857
**14. Sausages**	Turkey Sausage, Turkey Ham, Pork Sausage, Pork Ham, Chorizo	0.4851	
**15. Red Meat**	Barbacoa, Beef, Pork, Chicharron, Bacon		0.3861
**16. Sweet Bread**	Cake, Sweet Cookies and Bread, Filled Cupcakes, Hot Cake	0.5294	

**17. Industrialized Cereals**	Sweet Cereal, Cereal Bars, Sugar Free Cereal		
**18. Potato**	Potato	0.5973	
**19. Flours**	Bolillo, White bread, Pasta	0.424	
**20. Grains Cereals**	Whole Wheat Bread, Fiber Cereal, Oats		0.4515
**21. Mexican Dishes**	Torta, Tamale, Pozole, Tacos al pastor (Pork), Sopes, Carnitas (Pork)		
**22. Fast Food**	Burger, Hot dog, Pizza, Chicken Nuggets, Sandwich, French Fries	0.548	
**23. Salty Snacks**	Peanuts, Snacks, Crisps, Popcorn	0.434	
**24. Desserts**	Milkshake, Chocolate Milk, Danonino, Ice Cream, Chocolate, jam, Chamoy Candy		
**25. Dressings**	Dressing, Mayonnaise, Butter	0.5506	
**26. Sweet Beverages**	Cola Soft Drink, Fruit Soft Drink, Industrialized Juices, Hydrating Drinks, Water with Artificial Flavoring, Fruit Water		-0.4087
**27. Water**	Water	-0.7793	

Factor loadings or correlation coefficients between the variables (rows: groups of foods) and factors (columns: patterns 1 and 2).

Table S4: Dietary pattern score obtained for each child that participated based on their reported diet.

SampleID	Pattern1	Pattern2
**S117**	0.3205371	0.5758733
**S134**	0.1440915	-0.9681191
**S179**	-1.170841	-0.4113157
**S201**	0.9401742	0.143073
**S329**	0.1623228	-1.05331
**S339**	-0.0579411	-1.044442
**S402**	0.5130515	0.2638562
**S573**	-0.3296513	0.7141154
**S578**	3.497206	-0.5026956
**S847**	1.25227	0.5405591
**S865**	0.4539231	-0.2415238
**S884**	0.1391787	1.057557
**S1531**	-1.451602	0.5943944
**S1708**	-0.6210954	0.3915844
**S1914**	-0.0851043	-0.7586012
**S1922**	-0.6085021	2.942155
**S1969**	-0.0886021	2.483942
**S1972**	0.6682865	1.843158
**S2146**	0.6341589	-1.465647
**S2148**	0.2025058	0.9404573
**S2213**	2.288327	1.132702
**S2216**	2.511243	-0.397457
**S2221**	0.8065155	-1.299773
**S2269**	-0.3801049	0.3659545
**S2270**	-0.0431866	0.9992714
**S2289**	0.5796062	-0.3786825

**S2345**	-0.8007509	1.265548
**S2429**	0.4174601	0.4636037
**S2452**	-1.154805	-1.252507
**S2468**	-0.9809453	-0.2169433
**S2527**	0.0232354	-0.6029029
**S2593**	-0.5400318	-0.8162261
**S2600**	0.4270049	-0.4282541
**S2601**	-1.4277	0.4417136
**S2608**	-0.6509099	0.1371638
**S2627**	-0.75928	0.1697606
**S2632**	-0.6678567	-1.465489
**S2663**	-0.2033831	-0.4476009
**S2680**	-0.4065396	-0.310024
**S2726**	0.4214248	-1.822996
**S2817**	0.1120333	0.1744197
**S2846**	-0.1584052	0.3756168
**S2883**	-0.6839789	-0.9429005
**S2927**	-1.56976	-0.8547883
**S2967**	-0.6185966	0.125412
**S2971**	-1.05498	-0.4596928

Table S5. Relative abundance (>0.01%) of bacterial genera detected in fecal samples.

See file.

Table S6: Taxa (at genus or specie level) that contribute the most to beta diversity variation between NW and OWOB groups (SIMPER).

Taxa	Cumulative Relative Abundance	Taxa	Cumulative Relative Abundance	Taxa	Cumulative Relative Abundance
Prevotella copri	0.05639512	*[Eubacterium] siraeum*	0.43754507	*Bacteroides sp.*	0.59895415
Bacteroides dorei/vulgatus	0.09469867	*Ruminococcus sp. CAG:177*	0.45489865	*Clostridium sp. CAG:217*	0.61009154
Bacteroides stercoris	0.12424528	*Ruminococcus bicirculans*	0.46307388	*Bacteroides intestinalis***	0.62093356
Bacteroides rodentium/uniformis⁺	0.15170272	*Rikenellaceae sp.*	0.47121856	*Clostridium sp. CAG:343*	0.62623611
Bacteroides cellulosilyticus/fragilis	0.17562738	*Faecalibacterium prausnitzii*	0.48728578	*Faecalibacterium prausnitzii*	0.64193336
Eubacterium sp. CAG:202	0.19898182	*Barnesiella intestinihominis*	0.49515177	*Dehalococcoidales species incertae sedis*	0.63151148
*Eubacterium sp. CAG:180**	0.2209676	*Faecalibacterium sp.*	0.50239242	*Clostridium sp. CAG:127*	0.6367464
Bacteroides caccae	0.24095906	*Bacteroides caecimuris*	0.50961052	*Ruminococcaceae species incertae sedis*	0.64704771
Ruminococcus bromii	0.25849825	*Bacteroides plebeius*	0.51682632	*Roseburia inulinivorans*	0.65714674
Prevotella species incertae sedis	0.27540254	*Firmicutes sp.*	0.52392848	*Clostridium sp. CAG:465*	0.65211104
Alistipes putredinis	0.29225987	*Alistipes shahii*	0.5307857	*Clostridium sp. CAG:567*	0.66716776
Bacteroides sp.	0.30876689	*Bacteroides coprocola*	0.53762254	*Alistipes finegoldii*	0.67194751
Dialister invisus	0.32493854	*Parabacteroides merdae*	0.55100433	*Methanobrevibacter smithii**	0.67654813
Akkermansia muciniphila	0.33908238	*Bacteroides fragilis*	0.55758635	*Firmicutes species incertae sedis*	0.68109163
Bacteroides massiliensis	0.353011	*Faecalibacterium prausnitzii*	0.56388264	*Anaerotruncus sp. CAG:528*	0.68548795
[Eubacterium] rectale	0.36521315	*Clostridium sp. CAG:510*	0.57597579	*Bacteroides eggerthii*⁺	0.68972822
Clostridium sp. CAG:302	0.3768737	*Bifidobacterium longum*	0.58179301	*Roseburia species incertae sedis*⁺	0.69395928
Proteobacteria sp.	0.40918866	*Akkermansia species incertae sedis*	0.58756496	*Clostridiales species incertae sedis*	0.70223109

Alistipes finegoldii/onderdonkii	0.41935951	*Clostridium sp. CAG:594*	0.59331688

Cumulative contributions of most influential species are shown next to each genus. Pairwise comparisons using Wilcoxon rank sum test adjusted by FDR: $^{+}$: 0.1=>p>= 0.05, *: p <= 0.05, **: p <= 0.01

Table S7. KO representation analysis.

KO_entry	Name	Definition	logFC	logCPM	F	PValue	FDR	Differencially represented
**K16907**	K16907	fluoroquinolone transport system ATP-binding protein [EC:3.6.3.-]	4.402	5.316	20.845	0.000	0.002	UP
**K02838**	frr, MRRF, RRF	ribosome recycling factor	1.094	12.078	9.320	0.003	0.041	UP
**K02864**	RP-L10, MRPL10, rplJ	large subunit ribosomal protein L10	1.186	11.944	9.792	0.003	0.034	UP
**K14731**	mlhB, chnC	epsilon-lactone hydrolase [EC:3.1.1.83]	1.414	9.023	8.652	0.005	0.051	UP
**K00949**	thiN, TPK1, THI80	thiamine pyrophosphokinase [EC:2.7.6.2]	1.549	11.710	11.709	0.001	0.017	UP
**K09775**	K09775	uncharacterized protein	1.715	10.331	10.579	0.002	0.026	UP
**K01841**	pepM	phosphoenolpyruvate phosphomutase [EC:5.4.2.9]	1.793	8.446	8.914	0.004	0.045	UP
**K03366**	butA, budC	meso-butanediol dehydrogenase / (S,S)-butanediol dehydrogenase / diacetyl reductase [EC:1.1.1.- 1.1.1.76 1.1.1.304]	1.971	4.421	10.554	0.002	0.030	UP
**K19714**	kdnB	3-deoxy-alpha-D-manno-octulosonate 8-oxidase [EC:1.1.3.48]	2.022	4.427	10.660	0.002	0.029	UP
**K16872**	E2.3.1.207	beta-ketodecanoyl-[acyl-carrier-protein] synthase [EC:2.3.1.207]	2.080	4.435	10.793	0.002	0.028	UP
**K21400**	ortB	2-amino-4-ketopentanoate thiolase beta subunit [EC:2.3.1.263]	2.226	4.531	9.831	0.003	0.034	UP
**K18284**	K18284	adenosylhomocysteine/aminodeoxyfutalosine nucleosidase [EC:3.2.2.9 3.2.2.30]	2.291	4.467	11.953	0.001	0.018	UP
**K03821**	phbC, phaC	polyhydroxyalkanoate synthase subunit PhaC [EC:2.3.1.-]	2.307	4.468	11.498	0.001	0.021	UP
**K15363**	FAN1, MTMR15	fanconi-associated nuclease 1 [EC:3.1.21.- 3.1.4.1]	2.330	4.472	11.566	0.001	0.021	UP
**K15216**	RRN3, TIFIA	RNA polymerase I-specific transcription initiation factor RRN3	2.402	4.484	11.778	0.001	0.019	UP
**K00587**	ICMT, STE14	protein-S-isoprenylcysteine O-methyltransferase [EC:2.1.1.100]	2.473	4.497	12.945	0.001	0.014	UP

**K00814**	GPT, ALT	alanine transaminase [EC:2.6.1.2]	2.486	4.498	12.018	0.001	0.018	UP	
**K07110**	ramB	XRE family transcriptional regulator, fatty acid utilization regulator	2.487	4.498	12.022	0.001	0.018	UP	
**K23160**	wabB, rfbU	GlcNAc transferase	2.661	4.777	9.073	0.004	0.043	UP	
**K05499**	cytR	LacI family transcriptional regulator, repressor for deo operon, udp, cdd, tsx, nupC, and nupG	2.676	5.493	8.991	0.004	0.044	UP	
**K01228**	MOGS	mannosyl-oligosaccharide glucosidase [EC:3.2.1.106]	2.684	4.534	12.561	0.001	0.015	UP	
**K15311**	pksG	polyketide biosynthesis 3-hydroxy-3-methylglutaryl-CoA synthase-like enzyme PksG	2.722	4.544	12.737	0.001	0.015	UP	
**K03528**	zipA	cell division protein ZipA	2.803	5.809	9.673	0.003	0.036	UP	
**K13670**	pimF	putative glycosyltransferase [EC:2.4.-.-]	2.812	5.320	9.143	0.004	0.042	UP	
**K10917**	aphA	PadR family transcriptional regulator, regulatory protein AphA	2.844	4.941	9.720	0.003	0.035	UP	
**K22255**	ATPIF1	ATPase inhibitor, mitochondrial	2.857	4.572	13.606	0.001	0.011	UP	
**K10795**	prdD	D-proline reductase (dithiol)-stabilizing protein PrdD	2.877	4.778	11.360	0.001	0.019	UP	
**K00682**	GGCT	gamma-glutamylcyclotransferase [EC:4.3.2.9]	2.897	4.582	14.437	0.000	0.008	UP	
**K19713**	tsdA	thiosulfate dehydrogenase [EC:1.8.2.2]	2.966	4.719	11.890	0.001	0.016	UP	
**K21405**	acoR	sigma-54 dependent transcriptional regulator, acetoin dehydrogenase operon transcriptional activator AcoR	2.972	4.751	12.096	0.001	0.015	UP	
**K18652**	ntdC	glucose-6-phosphate 3-dehydrogenase [EC:1.1.1.361]	3.030	4.864	10.077	0.002	0.032	UP	
**K11940**	hspQ	heat shock protein HspQ	3.033	6.083	8.928	0.004	0.045	UP	
**K00143**	LYS2	L-2-aminoadipate reductase [EC:1.2.1.95]	3.050	4.618	13.564	0.001	0.011	UP	
**K14989**	salR	two-component system, NarL family, secretion system response regulator SalR	3.127	6.817	11.971	0.001	0.016	UP	

**K21006**	pelA	polysaccharide biosynthesis protein PelA	3.146	4.639	14.590	0.000	0.008	UP
**K13498**	trpCF	indole-3-glycerol phosphate synthase / phosphoribosylanthranilate isomerase [EC:4.1.1.48 5.3.1.24]	3.185	7.696	10.179	0.002	0.030	UP
**K01363**	CTSB	cathepsin B [EC:3.4.22.1]	3.188	4.647	13.794	0.001	0.010	UP
**K04646**	CLTC	clathrin heavy chain	3.239	4.660	13.908	0.001	0.010	UP
**K20179**	VPS11, PEP5	vacuolar protein sorting-associated protein 11	3.508	4.735	14.474	0.000	0.008	UP
**K23661**	escI	type III secretion system protein	3.599	4.766	15.272	0.000	0.006	UP
**K08568**	CTSZ	cathepsin X [EC:3.4.18.1]	4.262	5.005	15.800	0.000	0.005	UP
**K02656**	pilF	type IV pilus assembly protein PilF	4.837	5.269	17.925	0.000	0.003	UP
**K16906**	K16906	fluoroquinolone transport system permease protein	5.019	6.684	19.739	0.000	0.002	UP
**K15868**	baiB	bile acid-coenzyme A ligase [EC:6.2.1.7]	5.087	6.446	17.150	0.000	0.003	UP
**K16905**	K16905	fluoroquinolone transport system permease protein	5.387	6.998	20.569	0.000	0.002	UP
**K19161**	yafN	antitoxin YafN	5.831	7.197	27.412	0.000	0.000	UP
**K21488**	yobK	antitoxin YobK	-4.432	6.307	27.426	0.000	0.000	DN
**K01414**	prlC	oligopeptidase A [EC:3.4.24.70]	-5.392	7.108	32.822	0.000	0.000	DN
**K19266**	E1.2.1.22	lactaldehyde dehydrogenase [EC:1.2.1.22]	-4.321	4.859	23.561	0.000	0.002	DN
**K07728**	K07728	putative transcriptional regulator	-4.148	4.800	22.977	0.000	0.002	DN

**K18967**	dge1	diguanylate cyclase [EC:2.7.7.65]	-4.139	4.799	23.987	0.000	0.002	DN	
**K13942**	hmd	5,10-methenyltetrahydromethanopterin hydrogenase [EC:1.12.98.2]	-4.128	4.793	23.174	0.000	0.002	DN	
**K17717**	pld	phospholipase D [EC:3.1.4.4]	-3.987	4.753	24.440	0.000	0.002	DN	
**K10938**	acfC	accessory colonization factor AcfC	-3.933	4.744	21.642	0.000	0.002	DN	
**K06961**	KRR1	ribosomal RNA assembly protein	-3.923	4.865	25.111	0.000	0.001	DN	
**K11176**	purO	IMP cyclohydrolase [EC:3.5.4.10]	-3.869	4.715	22.134	0.000	0.002	DN	
**K03413**	cheY	two-component system, chemotaxis family, chemotaxis protein CheY	-3.794	11.959	34.839	0.000	0.000	DN	
**K09739**	mptD	dihydroneopterin aldolase [EC:4.1.2.25]	-3.768	4.687	21.772	0.000	0.002	DN	
**K23264**	purS	phosphoribosylformylglycinamidine synthase subunit PurS [EC:6.3.5.3]	-3.760	4.685	21.006	0.000	0.002	DN	
**K00368**	nirK	nitrite reductase (NO-forming) [EC:1.7.2.1]	-3.747	4.687	21.712	0.000	0.002	DN	
**K09139**	K09139	uncharacterized protein	-3.740	4.680	23.004	0.000	0.002	DN	
**K07072**	mfnF	(4-(4-[2-(gamma-L-glutamylamino)ethyl]phenoxymethyl)furan-2-yl)methanamine synthase [EC:2.5.1.131]	-3.729	4.677	21.931	0.000	0.002	DN	

**K19175**	dptH	DNA phosphorothioation-dependent restriction protein DptH	-3.713	5.080	18.077	0.000	0.003	DN
**K18974**	sul1	dihydropteroate synthase type 1 [EC:2.5.1.15]	-3.672	4.666	23.685	0.000	0.002	DN
**K22447**	cct, ths	archaeal chaperonin	-3.644	4.655	21.679	0.000	0.002	DN
**K01001**	ALG7	UDP-N-acetylglucosamine--dolichyl-phosphate N-acetylglucosaminephosphotransferase [EC:2.7.8.15]	-3.638	4.653	21.418	0.000	0.002	DN
**K00021**	HMGCR	hydroxymethylglutaryl-CoA reductase (NADPH) [EC:1.1.1.34]	-3.621	4.649	21.610	0.000	0.002	DN
**K18975**	ebr, qacEdelta1	small multidrug resistance pump	-3.617	4.653	23.157	0.000	0.002	DN
**K16509**	spxA	regulatory protein spx	-3.607	5.506	16.305	0.000	0.003	DN
**K22081**	mgsA	methylamine---glutamate N-methyltransferase subunit A [EC:2.1.1.21]	-3.557	4.634	21.277	0.000	0.002	DN
**K19824**	fprB	rubrerythrin	-3.516	4.624	21.241	0.000	0.002	DN
**K02910**	RP-L31e, RPL31	large subunit ribosomal protein L31e	-3.508	4.649	29.486	0.000	0.000	DN
**K02243**	comGA	competence protein ComGA	-3.455	4.775	20.232	0.000	0.002	DN
**K07765**	MBTPS2	S2P endopeptidase [EC:3.4.24.85]	-3.450	4.608	20.954	0.000	0.002	DN

**K01512**	acyP	acylphosphatase [EC:3.6.1.7]	-3.449	10.520	27.556	0.000	0.000	DN	
**K22223**	pgp	phosphoglycolate phosphatase [EC:3.1.3.18]	-3.428	4.885	16.593	0.000	0.003	DN	
**K14094**	ehaC	energy-converting hydrogenase A subunit C	-3.411	4.600	20.860	0.000	0.002	DN	
**K01170**	endA	tRNA-intron endonuclease, archaea type [EC:4.6.1.16]	-3.390	4.595	20.737	0.000	0.002	DN	
**K06875**	PDCD5, TFAR19	programmed cell death protein 5	-3.349	4.586	20.567	0.000	0.002	DN	
**K14096**	ehaE	energy-converting hydrogenase A subunit E	-3.316	4.579	20.489	0.000	0.002	DN	
**K12243**	pchR	AraC family transcriptional regulator, transcriptional activator of the genes for pyochelin and ferripyochelin receptors	-3.288	4.577	19.535	0.000	0.003	DN	
**K09738**	K09738	uncharacterized protein	-3.282	4.571	20.296	0.000	0.003	DN	
**K00340**	nuoK	NADH-quinone oxidoreductase subunit K [EC:7.1.1.2]	-3.262	11.259	40.744	0.000	0.000	DN	
**K02709**	psbH	photosystem II PsbH protein	-3.254	4.566	19.315	0.000	0.003	DN	
**K03167**	top6B	DNA topoisomerase VI subunit B [EC:5.6.2.2]	-3.250	4.760	17.187	0.000	0.003	DN	
**K22224**	acdB	acetate---CoA ligase (ADP-forming) subunit beta [EC:6.2.1.13]	-3.238	4.563	21.686	0.000	0.002	DN	

**K02966**	RP-S19e, RPS19	small subunit ribosomal protein S19e	-3.235	4.562	20.072	0.000	0.003	DN
**K14168**	CTU1, NCS6	cytoplasmic tRNA 2-thiolation protein 1 [EC:2.7.7.-]	-3.228	4.560	20.011	0.000	0.003	DN
**K03166**	top6A	DNA topoisomerase VI subunit A [EC:5.6.2.2]	-3.164	4.836	14.378	0.000	0.007	DN
**K00584**	mtrH	tetrahydromethanopterin S-methyltransferase subunit H [EC:2.1.1.86]	-3.151	4.918	12.927	0.001	0.011	DN
**K03299**	TC.GNTP	gluconate:H+ symporter, GntP family	-3.147	12.010	32.230	0.000	0.000	DN
**K00094**	E1.1.1.251, gatD	galactitol-1-phosphate 5-dehydrogenase [EC:1.1.1.251]	-3.141	4.795	16.076	0.000	0.004	DN
**K16793**	aksE	methanogen homoaconitase small subunit [EC:4.2.1.114]	-3.130	4.542	18.855	0.000	0.003	DN
**K14097**	ehaF	energy-converting hydrogenase A subunit F	-3.125	4.541	19.634	0.000	0.003	DN
**K17884**	E2.7.8.39	archaetidylinositol phosphate synthase [EC:2.7.8.39]	-3.121	4.539	19.607	0.000	0.003	DN
**K02530**	lacR	DeoR family transcriptional regulator, lactose phosphotransferase system repressor	-3.112	5.270	13.206	0.001	0.010	DN
**K06914**	mfnD	tyramine---L-glutamate ligase [EC:6.3.4.24]	-3.106	4.537	18.764	0.000	0.003	DN
**K12678**	aidA-I, misL	autotransporter family porin	-3.068	6.456	11.539	0.001	0.018	DN

**K14080**	mtaA	[methyl-Co(III) methanol-specific corrinoid protein]:coenzyme M methyltransferase [EC:2.1.1.246]	-3.063	4.529	19.389	0.000	0.003	DN
**K21306**	aglB	dolichyl-phosphooligosaccharide-protein glycotransferase [EC:2.4.99.21]	-3.048	4.526	18.539	0.000	0.003	DN
**K14095**	ehaD	energy-converting hydrogenase A subunit D	-3.029	4.523	19.194	0.000	0.003	DN
**K04480**	mtaB	methanol---5-hydroxybenzimidazolylcobamide Co-methyltransferase [EC:2.1.1.90]	-3.024	4.522	19.309	0.000	0.003	DN
**K16792**	aksD	methanogen homoaconitase large subunit [EC:4.2.1.114]	-3.021	4.521	19.340	0.000	0.003	DN
**K14109**	ehaR	energy-converting hydrogenase A subunit R	-2.993	4.516	19.122	0.000	0.003	DN
**K02929**	RP-L44e, RPL44	large subunit ribosomal protein L44e	-2.921	4.504	18.776	0.000	0.003	DN
**K09728**	K09728	uncharacterized protein	-2.921	4.504	18.857	0.000	0.003	DN
**K08093**	hxlA	3-hexulose-6-phosphate synthase [EC:4.1.2.43]	-2.914	5.319	10.351	0.002	0.029	DN
**K11600**	RRP41, EXOSC4, SKI6	exosome complex component RRP41	-2.899	4.500	18.724	0.000	0.003	DN
**K00537**	arsC	arsenate reductase (glutaredoxin) [EC:1.20.4.1]	-2.892	11.295	31.761	0.000	0.000	DN
**K14587**	sgcE	protein sgcE [EC:5.1.3.-]	-2.859	4.496	19.456	0.000	0.003	DN

**K04797**	pfdA, PFDN5	prefoldin alpha subunit	-2.838	4.490	18.453	0.000	0.003	DN
**K02877**	RP-L15e, RPL15	large subunit ribosomal protein L15e	-2.826	4.669	14.508	0.000	0.006	DN
**K04802**	PCNA	proliferating cell nuclear antigen	-2.825	4.488	18.225	0.000	0.003	DN
**K19665**	pssA	archaetidylserine synthase [EC:2.7.8.38]	-2.810	4.485	18.240	0.000	0.003	DN
**K00054**	mvaA	hydroxymethylglutaryl-CoA reductase [EC:1.1.1.88]	-2.796	5.064	9.879	0.003	0.034	DN
**K14108**	ehaQ	energy-converting hydrogenase A subunit Q	-2.795	4.483	18.293	0.000	0.003	DN
**K22579**	rph	rifampicin phosphotransferase [EC:2.7.9.6]	-2.788	4.718	13.348	0.001	0.010	DN
**K08971**	K08971	putative membrane protein	-2.743	4.475	17.292	0.000	0.004	DN
**K01058**	pldA	phospholipase A1/A2 [EC:3.1.1.32 3.1.1.4]	-2.743	9.597	16.817	0.000	0.003	DN
**K07101**	K07101	uncharacterized protein	-2.709	5.021	9.830	0.003	0.034	DN
**K11212**	cofD	LPPG:FO 2-phospho-L-lactate transferase [EC:2.7.8.28]	-2.705	4.681	11.986	0.001	0.016	DN
**K09776**	K09776	uncharacterized protein	-2.697	4.468	17.095	0.000	0.004	DN

**K00579**	mtrC	tetrahydromethanopterin S-methyltransferase subunit C [EC:2.1.1.86]	-2.694	4.595	14.487	0.000	0.006	DN
**K07463**	K07463	archaea-specific RecJ-like exonuclease	-2.677	4.539	17.236	0.000	0.003	DN
**K18814**	ictB	putative inorganic carbon (hco3(-)) transporter	-2.671	9.120	14.714	0.000	0.006	DN
**K18320**	IS15, IS26	transposase, IS6 family	-2.656	6.329	9.092	0.004	0.043	DN
**K07700**	dpiB, citA	two-component system, CitB family, cit operon sensor histidine kinase CitA [EC:2.7.13.3]	-2.640	5.191	9.174	0.004	0.042	DN
**K07161**	K07161	uncharacterized protein	-2.636	4.707	11.750	0.001	0.017	DN
**K19003**	mgdA	1,2-diacylglycerol 3-beta-glucosyltransferase [EC:2.4.1.336]	-2.621	5.599	9.305	0.003	0.041	DN
**K07799**	mdtA	membrane fusion protein, multidrug efflux system	-2.593	5.585	8.838	0.004	0.047	DN
**K13275**	isp	major intracellular serine protease [EC:3.4.21.-]	-2.573	4.453	17.292	0.000	0.004	DN
**K07739**	ELP3, KAT9	elongator complex protein 3 [EC:2.3.1.48]	-2.541	5.801	9.278	0.003	0.041	DN
**K22227**	ahbD	AdoMet-dependent heme synthase [EC:1.3.98.6]	-2.525	4.749	9.587	0.003	0.037	DN
**K22397**	yjhH, yagE	2-dehydro-3-deoxy-D-pentonate aldolase [EC:4.1.2.28]	-2.524	4.877	9.873	0.003	0.034	DN

**K07467**	rstA1	phage replication initiation protein	-2.488	11.141	22.980	0.000	0.002	DN
**K00442**	frhD	coenzyme F420 hydrogenase subunit delta	-2.468	4.476	18.383	0.000	0.003	DN
**K05937**	K05937	uncharacterized protein	-2.418	4.432	16.135	0.000	0.005	DN
**K09698**	gltX	nondiscriminating glutamyl-tRNA synthetase [EC:6.1.1.24]	-2.362	5.971	8.429	0.005	0.056	DN
**K03264**	EIF6	translation initiation factor 6	-2.348	4.608	9.930	0.002	0.034	DN
**K00320**	mer	5,10-methylenetetrahydromethanopterin reductase [EC:1.5.98.2]	-2.336	4.648	9.213	0.003	0.041	DN
**K07115**	rlmJ	23S rRNA (adenine2030-N6)-methyltransferase [EC:2.1.1.266]	-2.232	8.588	13.091	0.001	0.011	DN
**K02244**	comGB	competence protein ComGB	-2.167	4.578	9.505	0.003	0.038	DN
**K12234**	cofE, fbiB	coenzyme F420-0:L-glutamate ligase / coenzyme F420-1:gamma-L-glutamate ligase [EC:6.3.2.31 6.3.2.34]	-2.123	4.534	9.270	0.003	0.041	DN
**K00282**	gcvPA	glycine dehydrogenase subunit 1 [EC:1.4.4.2]	-1.817	7.858	8.540	0.005	0.053	DN
**K08591**	plsY	acyl phosphate:glycerol-3-phosphate acyltransferase [EC:2.3.1.275]	-1.589	11.848	17.741	0.000	0.003	DN
**K02860**	rimM	16S rRNA processing protein RimM	-1.554	12.344	16.752	0.000	0.003	DN

**K02794**	manX	mannose PTS system EIIAB component [EC:2.7.1.191]	-1.551	7.940	10.625	0.002	0.026	DN
**K02227**	cbiB, cobD	adenosylcobinamide-phosphate synthase [EC:6.3.1.10]	-1.546	10.824	20.197	0.000	0.002	DN
**K03215**	rumA	23S rRNA (uracil1939-C5)-methyltransferase [EC:2.1.1.190]	-1.544	12.041	16.374	0.000	0.003	DN
**K01704**	leuD, IPMI-S	3-isopropylmalate/(R)-2-methylmalate dehydratase small subunit [EC:4.2.1.33 4.2.1.35]	-1.541	11.589	16.409	0.000	0.003	DN
**K03402**	argR, ahrC	transcriptional regulator of arginine metabolism	-1.441	11.781	15.313	0.000	0.005	DN
**K00859**	coaE	dephospho-CoA kinase [EC:2.7.1.24]	-1.269	11.502	17.033	0.000	0.003	DN
**K07566**	tsaC, rimN, SUA5	L-threonylcarbamoyladenylate synthase [EC:2.7.7.87]	-1.165	12.161	12.485	0.001	0.013	DN
**K00873**	PK, pyk	pyruvate kinase [EC:2.7.1.40]	-1.115	12.469	12.037	0.001	0.016	DN
**K03702**	uvrB	excinuclease ABC subunit B	-1.098	12.530	11.622	0.001	0.018	DN
**K02224**	cobB-cbiA	cobyrinic acid a,c-diamide synthase [EC:6.3.5.9 6.3.5.11]	-1.061	10.660	9.888	0.003	0.034	DN
**K03615**	rnfC	Na+-translocating ferredoxin:NAD+ oxidoreductase subunit C [EC:7.2.1.2]	-1.020	11.800	9.345	0.003	0.040	DN

List of KOs differentially represented in the gut microbiome from NW vs. OWOB populations. (LogFC >1 and <-1, P and FDR adjusted-P <0.05).

Table S8: Enriched pathways as determined by Fisher's test.

path	allFreq	myFreq	pathInt	pathNoInt	backInt	backNoInt	pval	bonferroni	bh	Representation NW vs OWOB
ABC transporters	83	3	3	80	74	2774	0.37327499	1	0.46964551	over
Apoptosis	3	2	2	1	75	2853	0.00200939	0.0221033	0.00736777	over
Butanoate metabolism	24	2	2	22	75	2832	0.12979932	1	0.27068601	over
Chromosome and associated proteins	26	2	2	24	75	2830	0.14790293	1	0.27068601	over
Enzymes with EC numbers	122	4	4	118	73	2736	0.4000396	1	0.46964551	over
Exosome	24	3	3	21	74	2833	0.0237308	0.26103878	0.06525969	over
Lysosome	9	3	3	6	74	2848	0.00130709	0.01437797	0.00718899	over
Membrane trafficking	8	3	3	5	74	2849	0.00088814	0.00976954	0.00718899	over
Peptidases	55	2	2	53	75	2801	0.42695046	1	0.46964551	over
Transcription factors	83	4	4	79	73	2775	0.17225473	1	0.27068601	over
Transporters	284	3	3	281	74	2573	0.98421195	1	0.98421195	over
Chaperones and folding catalysts	31	3	3	28	166	2734	0.26382009	1	0.42320006	under
DNA repair and recombination proteins	46	2	2	44	167	2718	0.75407444	1	0.86578918	under
DNA replication proteins	15	3	3	12	166	2750	0.05133777	1	0.18308884	under
Energy metabolism	18	7	7	11	162	2751	3.47E-05	0.00107624	0.00053812	under
Enzymes with EC numbers	122	5	5	117	164	2645	0.84382502	1	0.90201985	under
Ether lipid metabolism	4	2	2	2	167	2760	0.01836051	0.56917579	0.11383516	under
Folate biosynthesis	15	2	2	13	167	2749	0.21290617	1	0.41250571	under
Function unknown	98	7	7	91	162	2671	0.33489834	1	0.45138472	under
Glycerolipid metabolism	15	2	2	13	167	2749	0.21290617	1	0.41250571	under
Glycerophospholipid metabolism	15	4	4	11	165	2751	0.0088208	0.27344465	0.07847071	under
Glycolysis / Gluconeogenesis	18	2	2	16	167	2746	0.27846123	1	0.42320006	under

Glycosyltransferases	24	3	3	21	166	2741	0.15738499	1	0.34849533	under
Glyoxylate and dicarboxylate metabolism	19	2	2	17	167	2745	0.30033553	1	0.42320006	under
Lipid biosynthesis proteins	17	2	2	15	167	2747	0.25654577	1	0.42320006	under
Lysine biosynthesis	10	2	2	8	167	2754	0.10969305	1	0.26157573	under
Methane metabolism	37	15	15	22	154	2740	4.33E-10	1.34E-08	1.34E-08	under
Nucleotide excision repair	5	2	2	3	167	2759	0.02944864	0.91290799	0.15215133	under
Peptidases	55	3	3	52	166	2710	0.62370322	1	0.80561666	under
Porphyrin and chlorophyll metabolism	31	3	3	28	166	2734	0.26382009	1	0.42320006	under
Prokaryotic defense system	45	2	2	43	167	2719	0.74305535	1	0.86578918	under
Purine metabolism	33	3	3	30	166	2732	0.29590391	1	0.42320006	under
Pyruvate metabolism	29	4	4	25	165	2737	0.08221778	1	0.21239593	under
Replication and repair	13	3	3	10	166	2752	0.03510594	1	0.15546916	under
Ribosome	26	4	4	22	165	2740	0.05906092	1	0.18308884	under
Ribosome biogenesis	33	6	6	27	163	2735	0.01012525	0.31388286	0.07847071	under
Secretion system	75	3	3	72	166	2690	0.81781991	1	0.90201985	under
Terpenoid backbone biosynthesis	8	2	2	6	167	2756	0.07357207	1	0.20733946	under
Transcription factors	83	4	4	79	165	2683	0.71554804	1	0.86578918	under
Transfer RNA biogenesis	37	5	5	32	164	2730	0.0586114	1	0.18308884	under
Transporters	284	7	7	277	162	2485	0.99825834	1	0.99825834	under
Two-component system	91	3	3	88	166	2674	0.90508105	1	0.93525042	under

Over-represented (over) and sub-represented (sub) KEGG pathways in the NW group compared to OWOB. *P-values* were adjusted by FDR according to Benjamini-Hochberg procedure.
