# Supplementary figures and images for: Alterations of the Gut Microbiome Associated to Methane Metabolism in Mexican Children with Obesity
Source: Children (Basel). 2022 Jan 24;9(2):148. doi: 10.3390/children9020148 (PMC8870140; doi:10.3390/children9020148)

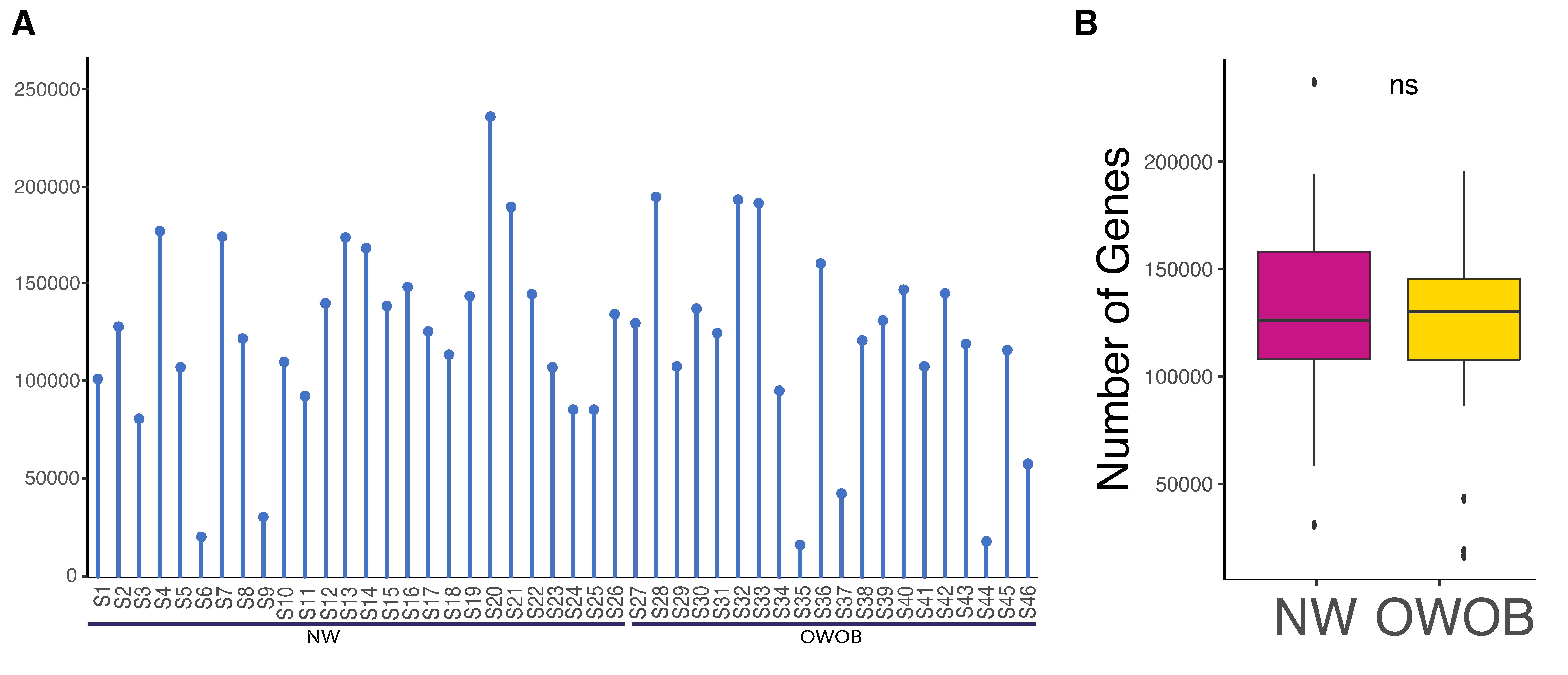

Supplement: Supplementary file 1 [file children-09-00148-s001.zip › Figure S1-Number of genes predicted for each library.tif]

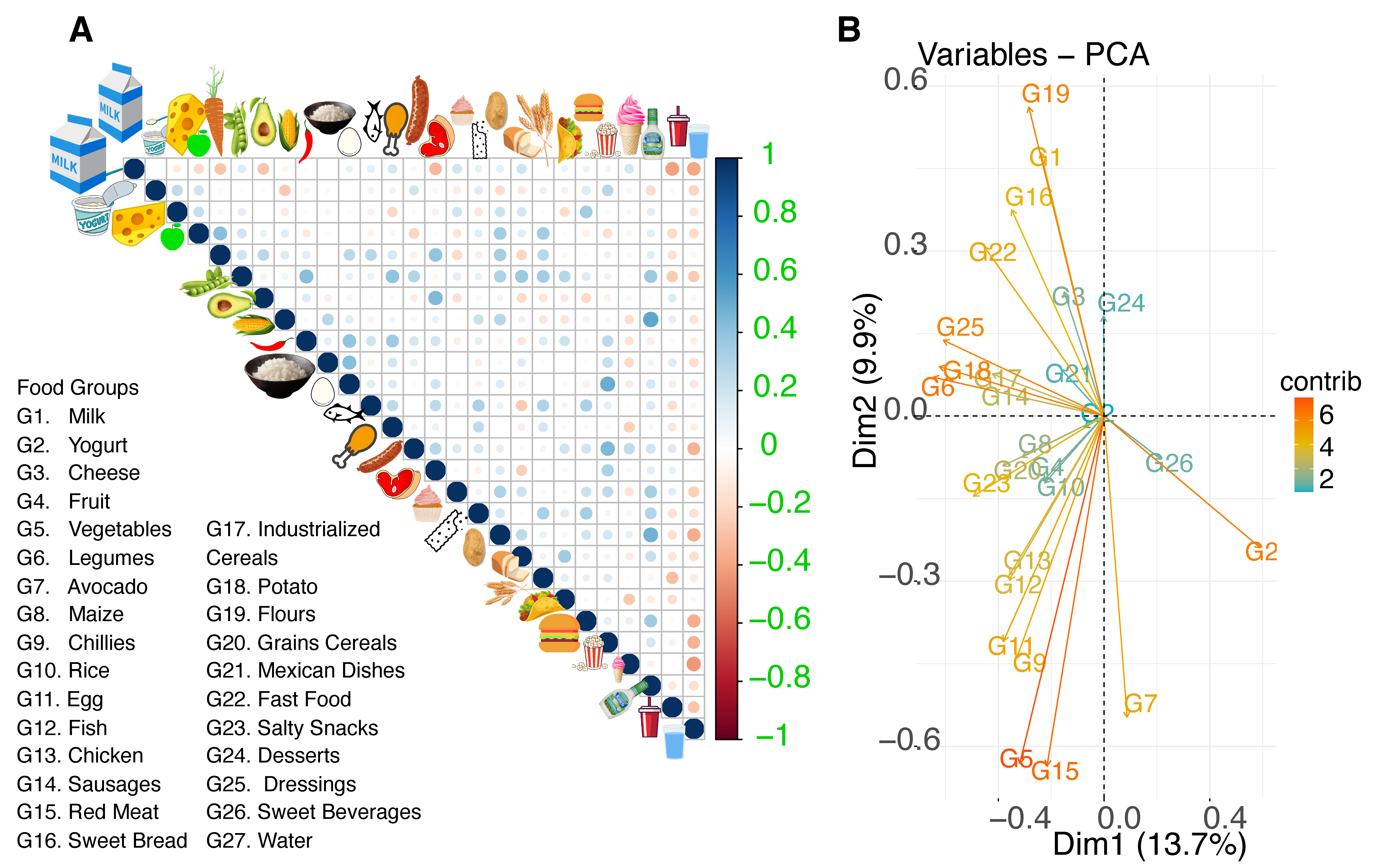

Supplement: Supplementary file 1 [file children-09-00148-s001.zip › Figure S2-Correlation matrix between food groups.tif]
